# Stimuli‐Responsive Hierarchical Structuring Controls Survival and Programs Hydrogelation of Supramolecular Metallofibers

**DOI:** 10.1002/adma.74151

**Published:** 2026-07-16

**Authors:** Merlin R. Stühler, László Mérai, Kai Ludwig, Rainer Haag, Quinn A. Besford, Alex J. Plajer

**Affiliations:** ^1^ Makromolekulare Chemie Universität Bayreuth Bayreuth Germany; ^2^ Institut Für Chemie und Biochemie Freie Universität Berlin Berlin Germany; ^3^ Leibniz‐Institut Für Polymerforschung e.V. Dresden Germany; ^4^ Bayerisches Polymer Institut (BPI) Universität Bayreuth Bayreuth Germany

**Keywords:** nanofiber, responsive polymer, smart hydrogels, stimuli responsive, supramolecular chemistry, supramolecular hydrogels, supramolecular polymers, thermo responsive

## Abstract

Hierarchical organization is a central strategy by which biological systems stabilize fragile assemblies and regulate function in hostile environments. Translating this principle to synthetic polymer systems in water remains challenging, as noncovalent interactions can readily disrupt supramolecular polymerization. Herein, we report on aqueous supramolecular metallofibers formed by Zn(II) Salphen complexes functionalized with poly(*N*‐isopropylacrylamide) (PNIPAM), which undergo reversible, multi‐stimuli‐responsive hierarchical structuring. Individual nanofibers form spontaneously in dilute aqueous solution via supramolecular copolymerization with water molecules and reversibly organize into micron‐sized bundles in response to temperature, ionic strength, and solvent composition. This higher‐order organization kinetically stabilizes the assemblies under conditions of dilution, low pH, and chemical degradation, where both monomers and isolated nanofibers rapidly disassemble. Hierarchical structuring further modulates photophysical properties, enables selective sorting and survival of responsive over non‐responsive fibers in mixed systems, and translates microscopic robustness into macroscopic hydrogelation at unusually low concentrations. Leveraging the intrinsic photothermal response of the Zn(II) Salphen chromophore, light irradiation drives an energy‐dependent, out‐of‐equilibrium bundled state that persists only while light energy is supplied, thereby controlling chemical stability in acidic environments. Collectively, this work establishes hierarchical supramolecular structuring in water as a general strategy to program stability, selectivity, and energy‐dependent function in synthetic soft materials.

## Graphical abstract

Aqueous Zn(II) Salphen metallofibers decorated with PNIPAM undergo reversible, multi‐stimuli‐responsive hierarchical bundling. This higher‐order structuring kinetically stabilizes the otherwise fragile assemblies against dilution, acidic hydrolysis, and transmetallation, enables selective sorting of responsive fibers, and programs hydrogelation at unusually low concentrations. Light sustains an out‐of‐equilibrium bundled state controlling chemical stability.

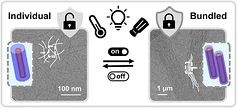

## 1 Introduction

One of the most fundamental organizational principles in living systems is the formation of functional supramolecular assemblies, which serve as essential platforms enabling cells to maintain structural integrity, sense environmental changes, and execute dynamic cellular processes [1, 2]. The cell cytoskeleton exemplifies this principle through its one‐dimensional supramolecular assemblies (particularly actin filaments and microtubules) which achieve highly ordered and dynamic organization through reversible, noncovalent interactions rather than permanent chemical bonds [3, 4, 5]. Central to the biological effectiveness of these systems is their ability to operate far from thermodynamic equilibrium in environments where formation and stability are governed kinetically [6, 7]. Moreover, biological systems show the capacity to selectively sort, position, and hierarchically organize supramolecular cytoskeletal structures to encode distinct functionalities such as force generation, intracellular transport, and signal integration. Such selective compartmentalization not only enables the concentration of functional molecules and maintains chemical gradients but also protects fragile assemblies from disruptive chemical stimuli which is fundamental to the origin and persistence of life [8, 9, 10, 11].

While synthetic supramolecular polymers typically lack the complexity and dynamic functionality of biological systems, they can be engineered to controllably and selectively assemble into hierarchical structures in response to external stimuli [12, 13, 14, 15]. Several studies demonstrated that higher‐order structuring of supramolecular fibrous architectures can be induced by various stimuli, such as solvent switches, X‐ray irradiation, or molecular crowding [16, 17, 18]. Higher‐order assembly, for example, can be tuned to generate distinct topologies, such as ribbons and Möbius strips [19]. However, these kinetically controlled processes were inherently unidirectional, and the resulting structures remained non‐functional. By contrast, Stupp and coworkers demonstrated that, using naturally occurring supramolecular motifs in water, hierarchical structure formation could be reversibly controlled by altering the chemical composition of the medium, rather than presenting a one‐way aggregation process [20]. Nevertheless, it remained unclear to what extent hierarchical structuring influences the chemistry of the individual components, or whether it can be harnessed to sort supramolecular structures and construct functional materials with bioinspired enhanced robustness [21, 22, 23, 24, 25].

Water is arguably the most relevant medium to achieve supramolecular self‐assembly and polymerization [26, 27, 28, 29, 30, 31, 32, 33]. This can, however, be challenging due to the disruption of noncovalent interactions with surrounding water molecules, meaning that interaction sites for self‐assembly often have to be shielded from the aqueous medium, which at most interacts with the exterior of the assemblies [34, 35, 36].

In the context of aqueous self‐assembly, we recently reported that specific zinc(II) bisphenoxyimine (“Salphen”) complexes functionalized with solubilizing polyethylene glycol (PEG) chains undergo supramolecular polymerization in water to form tubular nanofibers [37]. This arises from an unconventional assembly logic in which water molecules act as structural co‐monomers rather than exclusively being excluded via the hydrophobic effect. This contrasted with previous reports, which either achieved Salphen self‐assembly or copolymerization of water, in organic or solvent‐free systems [38, 39, 40, 41, 42, 43, 44, 45, 46, 47].

In principle, these fibers could be used as a platform for constructing stimuli‐responsive hierarchical structuring by replacing the PEG appendages. In this regard, poly(*N*‐isopropylacrylamide) (PNIPAM) undergoes conformational collapse with increased adhesion in response to changes in temperature and the chemical nature of the surrounding medium [48, 49, 50]. Accordingly, we hypothesized that conjugation of PNIPAM onto the monomeric units might be suitable to impart this functionality onto the supramolecular metallofibers.

## 2 Results and Discussion

We first synthesized monomer **A^o^** (Figure 1a, Section S02), in which the *ortho*‐methoxy‐substituted Zn(II) Salphen based assembly unit was connected to PNIPAM via amide coupling (*M*_n_ (PNIPAM) = 2500 Da). Furthermore, we also prepared derivatives of **A^o^** bearing methoxy groups in the *meta*‐ (**A^m^**) and *para*‐positions (**A^p^**) via the same route, as supramolecular copolymerization of water is crucially influenced by the exact geometry of the water coordination site of the Salphen core.

**FIGURE 1:**
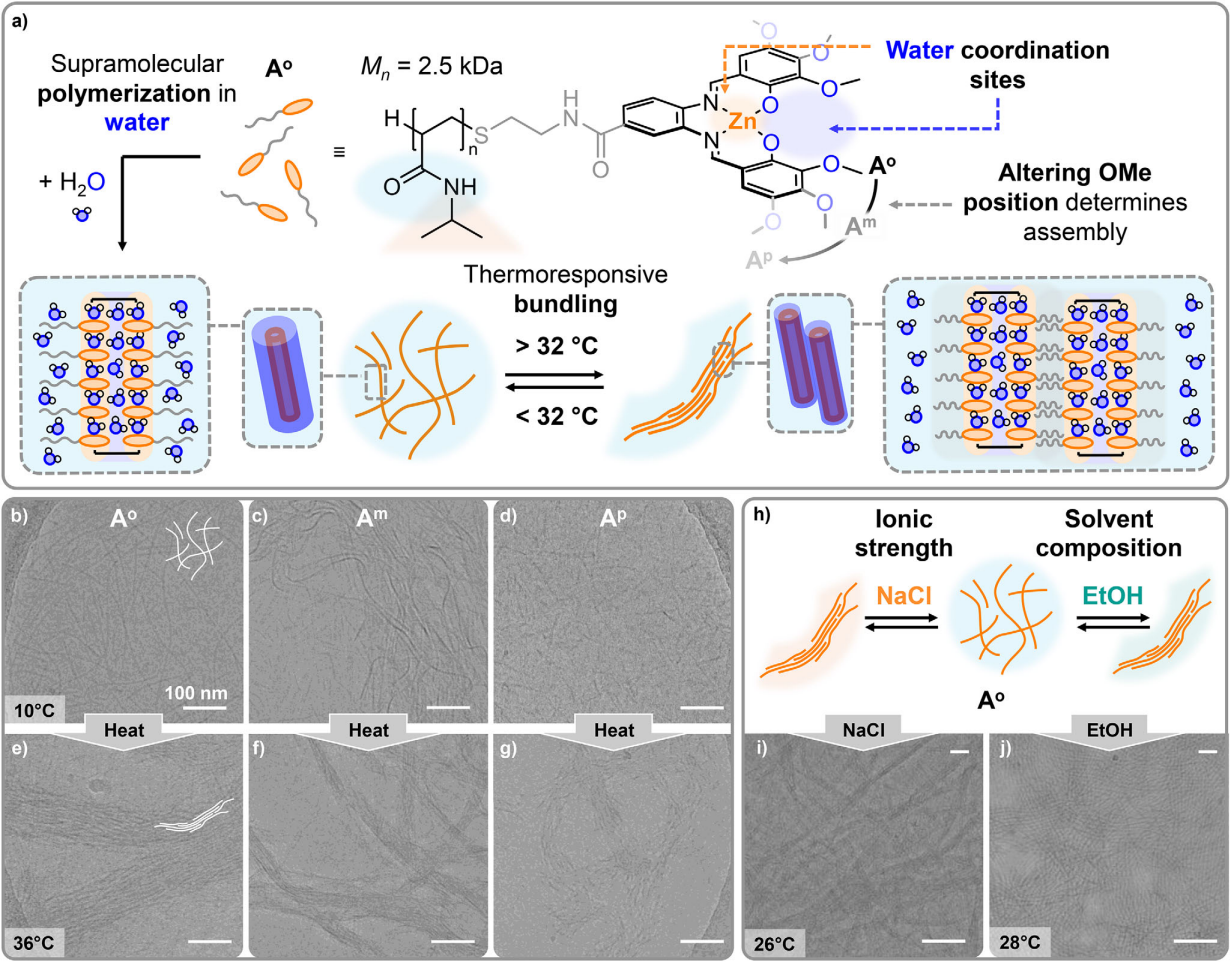
(a) Chemical structure of A^o^, A^m^, and A^p^ (o: *ortho*, m: *meta*; p: *para*), respectively, and schematic representation of the thermoresponsive bundling of A^o^. (b‐d) Cryo‐TEM images (28 000x) of 0.1 mM A^o^, A^m^ and A^p^, respectively, vitrified at 10°C. (e‐g) Cryo‐TEM images (28 000x) of 0.1 mM A^o^, A^m^ and A^p^, respectively, vitrified at 36°C. (h) Schematic representation of the bundling of A^o^ triggered by increased ionic strength or solvent composition. (i, j) Cryo‐TEM images (28 000x) of 0.1 mM A^o^ vitrified at 26°C in 0.5 M NaCl and 28°C in 15 % EtOH solution, respectively.

The cryogenic transmission electron microscopy (cryo‐TEM) image of a 0.1 mM aqueous solution of **A^o^** at 10°C reveals the formation of uniform fibers in the range of hundreds of nanometers (Figure 1b). Interestingly, while **A^p^** shows ineffective fiber formation, **A^m^** forms ribbon‐like aggregates under the same conditions, as further confirmed by cryogenic electron tomography (cryo‐ET) (see Figure 1c,d and Figures S13 and S14). In reference to our recent work, this dependence of the supramolecular architecture and resulting morphology on the position of the methoxy substituents confirms assembly of the PNIPAM‐decorated metallofibers via supramolecular copolymerization of water [37]. Notably, two hierarchical levels of self‐assembly are present: one‐dimensional supramolecular stacking, which is most efficient for **A^o^** but also pronounced for **A^m^**, and lateral association of the resulting stacks into higher‐order assemblies, where the methoxy substitution pattern becomes morphology‐determining, yielding either fibrous (**A^o^**) or ribbon‐like (**A^m^**) structures.

Stand‐alone PNIPAM of similar molecular weight forms uncontrolled aggregates above 32°C—its lower critical solution temperature (LCST)—where it undergoes a coil‐to‐globule transition by expelling solubilizing water molecules and collapsing [51]. However, the well‐defined geometry of the PNIPAM‐decorated metallofibers and ribbons guides this aggregation laterally. Cryo‐TEM examinations of **A^o^**, **A^m^**, and **A^p^** at 36°C revealed the induction of morphological changes at the microscopic scale (Figure 1e–g) as the previously independent fibers start to bundle. Note that vitrification of the aqueous samples for cryo‐TEM analysis occurs so rapidly that the system does not have sufficient time to relax into its room‐temperature morphology beforehand. For **A^o^** and **A^m^**, these bundles far exceed the size of single holes of the holey carbon grid (R1.2/1.3) and form an interconnected network spanning across the entire grid mesh (see Figures S12 and S15, respectively). The bundles of **A^o^** thereby appear larger than those of **A^m^**. The rudimentary assemblies of **A^p^**, on the other hand, bundle into ill‐defined aggregates (see Figure S17). We previously showed that placing Ni(II) instead of Zn(II) within the Salphen scaffold does not lead to supramolecular copolymerization with water due to its reduced Lewis acidity and tendency for axial coordination. Accordingly, **A^Ni^**, the Ni(II) version of **A^o^**, which is prepared with Ni(OAc)_2_ in place of Zn(OAc)_2_, also does not undergo regular hierarchical structure formation. Taken together, it becomes evident that by strategically modulating the Zn(II) Salphen assembling units, not only morphological changes on the single fiber but also on the hierarchical structure level can be achieved. Most importantly, bundling triggered by LCST‐induced phase separation is fully reversible, and at room temperature, the micron‐sized bundles disentangle into individual fibers that are an order of magnitude smaller, reverting the assembly to the nanoscale. Thereby, our system allows for rapid and dynamic control of aqueous self‐assembly across multiple lengths scale.

Chemical stimuli can likewise induce hierarchical structuring. Accordingly, we used NaCl as a chaotropic salt, which “disrupts” the water structure and weakens the hydration layer, making the PNIPAM polymer shell collapse and thus trigger bundle formation below the typical phase‐transition temperature of PNIPAM in pure water [52]. Cryo‐TEM analysis of **A^o^** in 0.5 M NaCl solution at 26°C indeed shows substantial bundling of the nanofibers (Figure 1h), conditions in which no hierarchical structuring occurs in pure water. Similarly, the addition of certain organic solvents, such as ethanol, can bundle the PNIPAM‐functionalized nanofibers via the co‐non‐solvency effect (PNIPAM is soluble in pure good solvents, but insoluble in a mixture of two good solvents, such as EtOH in water, in the water‐rich regime) [49]. Cryo‐TEM in 15 vol% EtOH at 28°C, a temperature at which no bundling occurs in pure water, reveals higher‐order aggregates far exceeding the nanoscopic scale of the individual fibers. Note that bundles induced by ionic strength and solvent composition appear more diffuse on the micrometer scale, which might be a result of lowered vitrification efficiency during the grid preparation process of the cryo‐TEM measurement, and thus influenced the environment of the fibers during sample preparation. With the knowledge gained about the versatile triggers, we were interested in examining the consequences arising from bundle formation beyond morphological changes.

We hypothesized that the hierarchical structuring maintains the aggregates in a kinetically trapped state protecting them from dissociation upon dilution. Therefore, we investigated the dilution stability of the nanofibers of **A^o^** in the detached (below LCST) as well as the bundled state (above LCST) via dynamic light scattering (DLS) (see Figure 2a). The critical aggregation concentration (CAC), being the threshold concentration below which the fibers are fully depolymerized into monomeric complexes, was found to be 1.1 ± 0.2 µM for the individual fibers at 25°C. When the solution, however, is at a temperature of 40°C, the bundling effectively prevents disassembly upon dilution, thereby lowering the CAC by 50‐fold to 20 ± 0.7 nM. Thus, bundled fibers are stable at concentrations at which individual fibers would otherwise be unstable.

**FIGURE 2:**
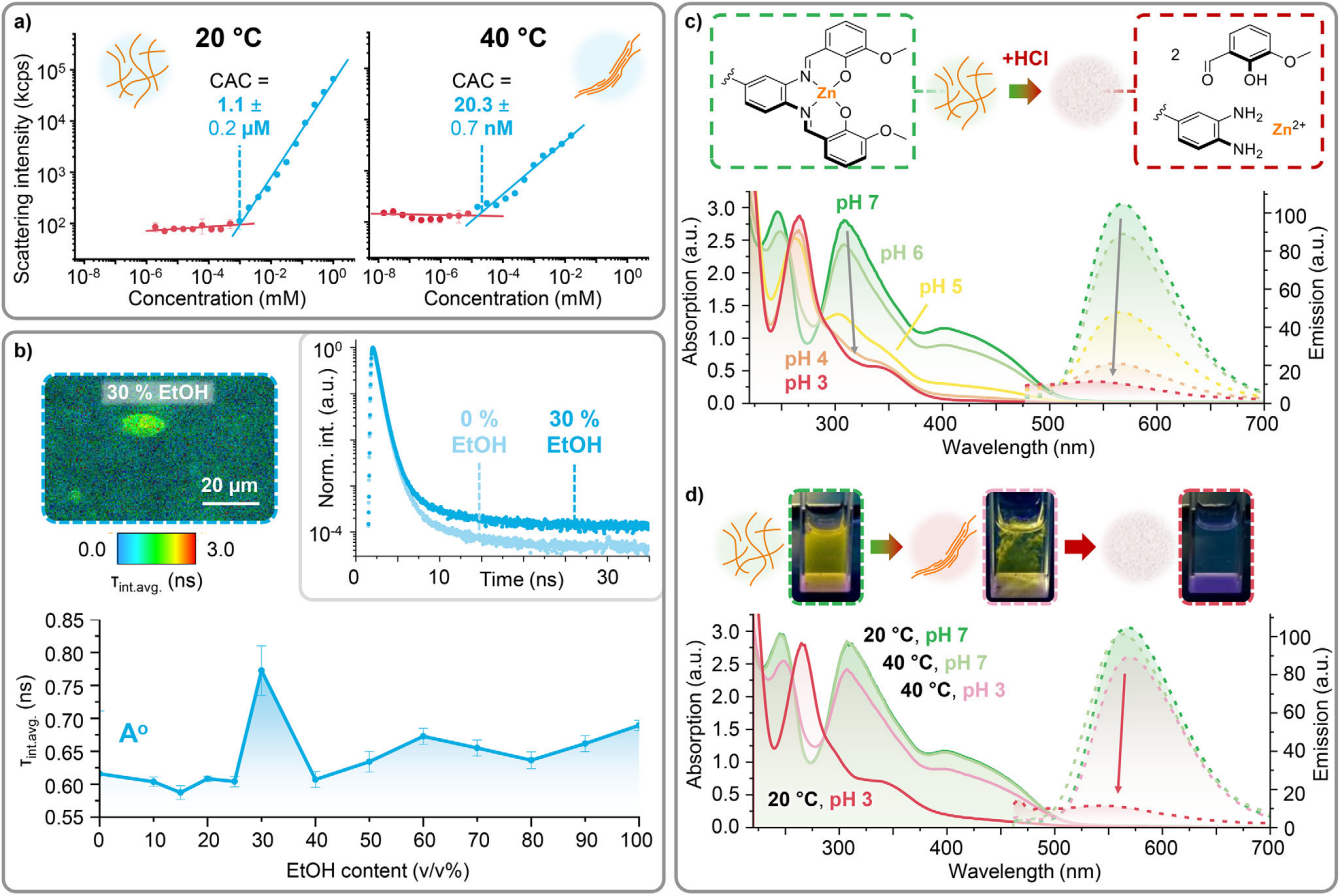
(a) Critical aggregation concentration (CAC) of **A^o^** at 20°C and 40°C, respectively, determined by DLS. (b) Fluorescence lifetime of 0.1 mM **A^o^** in water/EtOH mixtures as a function of EtOH content (*λ*_exc._= 405 nm, *λ*_cutoff_ < 425 nm) with FLIM heatmap at 30 vol% EtOH. The inlayed graph shows the TCSPC at 0 vol% and 30 vol% EtOH content. UV–Vis absorption (—) and photoemission (‐ ‐) spectra of a 100 µM solution of **A^o^**: (c) measured from pH 7 (green) to pH 3 (red) at 20°C, showing acid‐catalyzed hydrolysis; and (d) measured at pH 7 and 20°C, then heated to 40°C, which suppressed hydrolysis upon subsequent acidification to pH 3, followed by cooling to 20°C, where hydrolysis finally occurred including corresponding photographs of the cuvette.

Questioning whether the morphological changes also influence molecular electronic properties, we used fluorescence lifetime imaging microscopy (FLIM) to investigate the differences in fluorescence lifetime in dependence of solvent composition (see Section S04). This allowed us to spatially resolve the higher‐order aggregates through increased fluorescence lifetime. Here, a peak increase of approximately 30 % was observed at 30 vol% EtOH for **A^o^** at 1 mM concentration along with pronounced aggregate formation—indicative of aggregation‐induced emission (Figure 2b) [53, 54]. This is in alignment with the co‐non‐solvency regime of PNIPAM and the bundled state of the fibers. Here, the time‐correlated single‐photon counting (TCSPC) further showed a distinct offset in fluorescence lifetime (see inset of Figure 2b). With ethanol content raised beyond the co‐non‐solvency regime, PNIPAM re‐expands, bundle structures disentangle, and the fluorescence lifetime correspondingly declines. Moreover, the FLIM images of **A^m^** showed microscopic aggregation along with a lifetime peak in a similar regime (15–40 vol%). Importantly, **A^p^**, which does not undergo regular hierarchical structuring, did not show any measurable fluorescence increase associated with aggregation. This confirms that bundling enhances properties down to the molecular electronic level, and controlled morphology formation is central to this effect.

The Salphen ligand and its metal complexes are susceptible to acidic hydrolysis of the imine functionalities. Accordingly, we wanted to assess the protective power of hierarchical structuring against acids representing a hostile chemical environment. For monitoring, we utilized UV–Vis and photoemission spectroscopy as the Salphen complex shows distinct bands compared to its hydrolyzed aldehyde and amine components.

At 20°C, a 100 µM aqueous solution of **A^o^** showed absorption peaks around 248 and 310 nm as well as pronounced photoemission at 560 nm as characteristic for Zn(II) Salphen chromophores (Figure 2c). Upon acidification at 20°C, decomposition of the fibers and monomers at pH ≤ 5 was observed as indicated by a shift of the absorption profile to a single peak at 266 nm and nearly complete loss of photoemission. Strikingly, when the solution was heated to 40°C before acidification, the absorption profile as well as the photoemission intensity remained mostly stable and only when the solution was cooled down to 20°C, decomposition occurred as shown in Figure 2d. This suggests that a substantial fraction of the fibers (approx. 90 %) is incorporated into bundles, which provide a protective environment against acidic hydrolysis by effectively shielding the fibers from the harsh conditions. However, while the PNIPAM chains on the exterior become hydrophobic, the internal structural water molecules remain incorporated, as evidenced by the preserved fibrous morphology of the assemblies upon bundling. As this shielding effect is specific to the polymer shell of the fibers, it suggests the possibility of selective hierarchical ordering in more complex mixtures of fibers with and without responsive shells. To demonstrate this concept, we resorted to the mPEG‐functionalized nanofibers (**B^o^**), lacking the potential for higher‐order structuring, and mixed aqueous assemblies of **A^o^** and **B^o^** in a 1:1 ratio. We employed monomers bearing mPEG *M_w_* = 500 Da, as higher‐molecular‐weight mPEG chains have been found to compromise the stability of the resulting assemblies [37]. Upon heating, **A^o^** selectively sorts into bundles in the presence of non‐aggregating fibers of **B^o^**. Cryo‐TEM images at 36°C nicely reveal the co‐existence of both microscopic bundles of **A^o^** and individual fibers of **B^o^** as depicted in Figure 3a. If bundling enhances stability, only bundles should survive acidification. Accordingly, as the mixed‐assembly solution was heated to 40°C and subsequently acidified, both the UV–vis and photoemission profiles showed a 50 % reduction of the absorption and emission peaks corresponding to the intact Zn(II) Salphen chromophores, implying that only the accessible fibers of **B^o^** were decomposed while the resistant bundles of **A^o^** endured (Figure 3b). Upon cooling of the mixture and thus release of the bundled fibers of **A^o^**, the absorption and photoemission profile of the Zn(II) Salphen units fully vanished.

**FIGURE 3:**
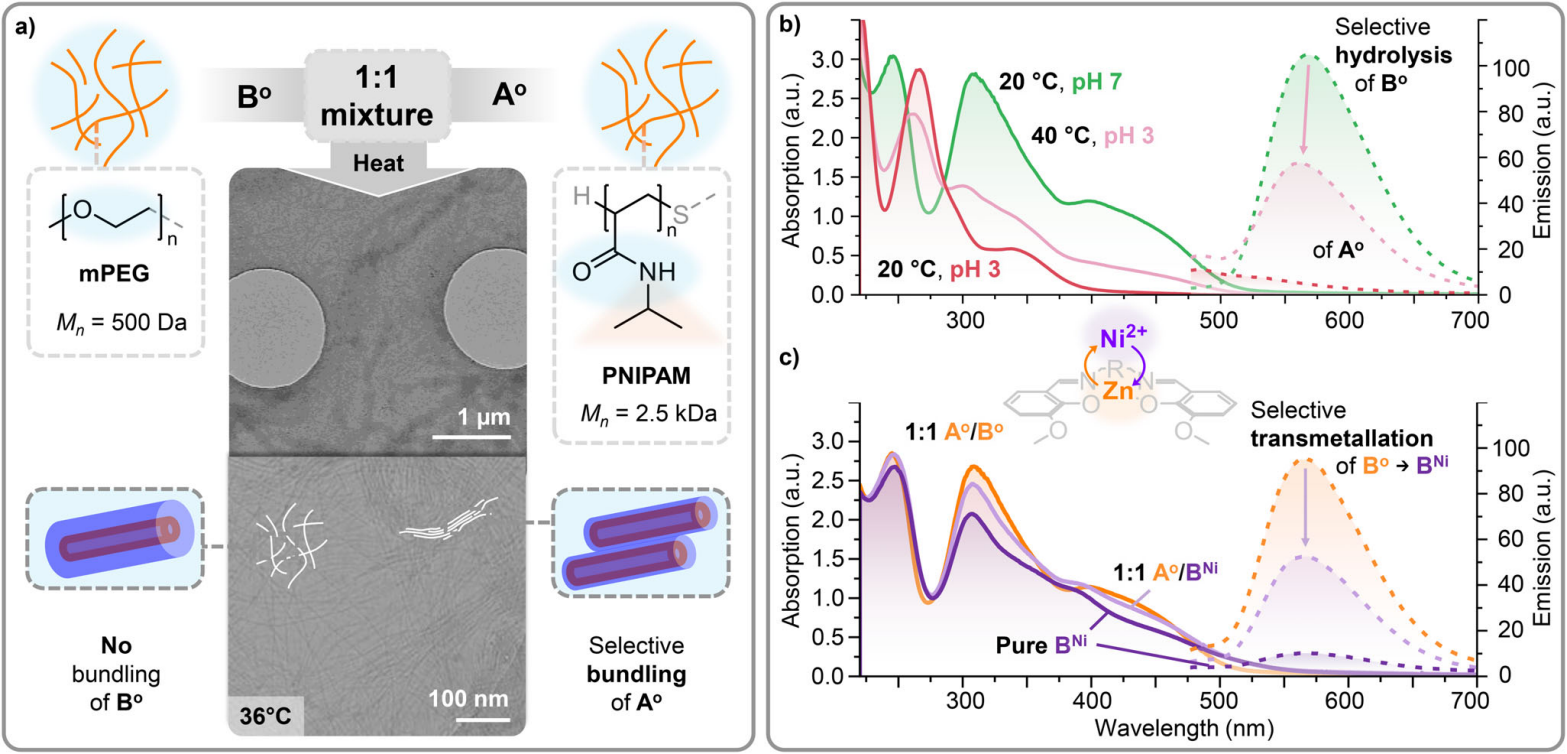
(a) Cryo‐TEM images (3400x and 28000x) of **A^o^** and **B^o^**, respectively, vitrified at 36°C. UV–vis absorption (—) and photoemission (‐ ‐) spectra of a 100 µM 1:1 mixture of **A^o^** and **B^o^**: (b) measured at 20°C, pH 7, 40°C, pH 3 and 20°C, pH 3, showing selective hydrolysis of **B^o^**; and (c) measured at 20°C and after addition of Ni(OAc)_2_ and heating to 90°C showing selective transmetallation of **B^o^** as well as the spectrum of a transmetallated 100 µM solution of **B^o^** only.

But not only acidic environments pose a threat for the nanofibers in solutions, as competitive metal ions, such as Ni(II) can exchange the Zn(II) center in the Salphen ligand, driven by strong ligand field stabilization of Ni(II), leading to dissociation of the fibers [53, 55]. This transmetallation reaction, however, needs thermal activation and thus only occurs at elevated temperatures. Accordingly, we tested whether the bundle formation could also protect the Zn(II) Salphen core units of **A°** from transmetallation with Ni(II) which would effectively transform it into **A^Ni^**. Therefore, Ni(OAc)_2_ was added to a 1:1 mixture of **A^o^** and **B^o^**, and the solution was subsequently heated to 90°C to thermally activate the transmetallation process. UV–vis absorption and emission spectroscopy revealed that the transformation proceeded to only ∼50 %, yielding spectra intermediate between those of the starting mixture and the characteristic profiles of the corresponding Ni(II) Salphen complex **B^Ni^**, as confirmed by comparison with a fully transmetallated solution of **B^o^** (see Figure 3c). Taken together, this revealed that the controlled hierarchical structuring can sort assemblies and selectively protect the molecular assembling units from harsh chemical environments.

Having understood the hierarchical self‐assembly of **A^o^** on the nano‐ and microscopic scale, we aimed for utilizing bundling on the macroscopic material level. Interestingly, we observed hydrogelation when heating aqueous solutions of **A^o^**, which reverted upon cooling to reform a liquid (Figure 4a). Accordingly, variable‐temperature (VT) rheology of a 3 mM aqueous solution shows an abrupt increase in *G’* from 1 to 1023 Pa at 32°C and, upon cooling to room temperature, decreases again to 5 Pa. Note that stand‐alone PNIPAM only forms hydrogels at very high molecular weights (∼10^5^–10^7^ g/mol) and concentrations exceeding 7 wt% via entanglement [56, 57]. Hydrogelation of **A°** featuring comparatively short PNIPAM chains of ∼10^3^ g/mol, however, already occurs at concentrations as low as 500 µM—about a 200‐fold reduction in concentration (see Figure S25). Varying the molecular weight of the coupled PNIPAM to 5.5 kDa leaves the gelation‐onset temperature largely unchanged and only leads to a slightly steeper increase in the mechanical moduli upon hydrogelation compared to 2.5 kDa PNIPAM‐**A^o^** (see Figure S26). This can be rationalized by the hierarchical nature of the nanofibers: a supramolecular polymer composed of multiple monomeric units, each bearing short PNIPAM chains, effectively behaves as an ultra‐high‐molecular‐weight PNIPAM system, making differences in individual side‐chain molecular weight largely negligible. Furthermore, the isomers **A^m^** and **A^p^** showed distinct behaviors upon heating (Figure 4a). For **A^p^**, rheology only showed a minimal increase in *G’* when reaching the LCST of PNIPAM, and no hydrogelation occurs. **A^m^**, on the other hand, shows an increase in *G’* from 1 to 275 Pa and hence reversibly forms a hydrogel at 3 mM. This is in line with the microscopic bundling capabilities of the investigated monomers and the fact that **A^m^** forms less extended hierarchical structures than **A^o^**. The controlled and immediate hydrogelation of **A^o^** was leveraged for the controlled extrusion of a gel string into water at 40°C (Figure 4b). Subsequent cooling to room temperature resulted in complete dissolution of the gel.

**FIGURE 4:**
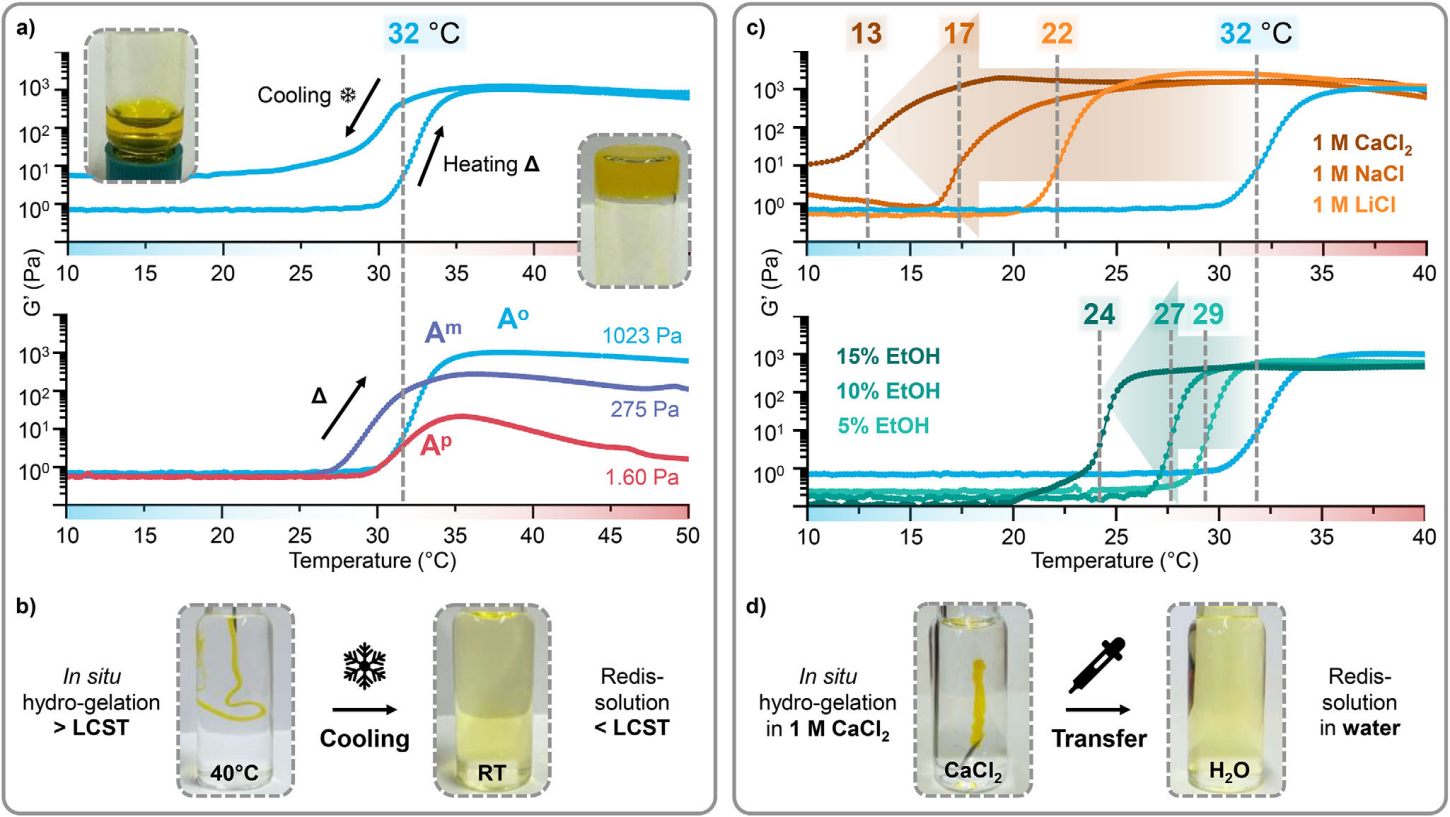
(a) (top) Temperature‐dependent rheology curve of A^o^ in water, including photographs of a 3 mM solution of A^o^ below and above the LCST, as well as (bottom) rheology curves of A^o^, A^m^, and A^p^, respectively. (b) Photographs of an in situ formed hydrogel by injection (0.1 mL/min) of a 3 mM solution of A^o^ into 40°C water and subsequent re‐dissolution upon cooling to room temperature. (c) Temperature‐dependent rheology curves of A^o^ in water and 1 M solutions of chaotropic salts with increasing ionic strength (LiCl, NaCl, CaCl_2_; top) as well as solvent mixtures of water with 5–15 vol% EtOH (bottom). (d) Photographs of an in situ formed hydrogel by injection (3 mL/min) of a 3 mM solution of A^o^ into 1 M CaCl_2_ solution and subsequent re‐dissolution upon transfer into water.

As the bundling can be triggered by chemical stimuli, we were able to tune the rheological profiles of the hydrogel by ionic strength and solvent composition. VT‐rheology revealed precise control over the hydrogelation temperature by either using chaotropic salts or varying amounts of EtOH (Figure 4c). For example, the hydrogelation temperature of **A^o^** was progressively decreased from 32°C in pure water to 22°C in LiCl, 17°C in NaCl and 13°C in CaCl_2_ (all at 1 M concentration). Hence, the most chaotropic salts decreased the LCST below room temperature so that at ambient conditions a stable hydrogel formed. Accordingly, extrusion of 3 mM **A^o^** into a 1 M CaCl_2_ solution at room temperature led to immediate hydrogelation. When the ionic strength was lowered, e.g. by transferring the gel string into pure water, it entirely re‐dissolved again (Figure 4d). Further tuning of the gelation temperature was also achieved by varying both the salt concentration and, in agreement with the Hofmeister series, the anion identity (Cl^−^ < I^−^ < SCN^−^) (see Figures S27 and S28, respectively). Taken together, these results highlight **A^o^** as a potential recoverable 3D printing resin, combining low material requirements with fully reversible, multi‐stimuli‐controlled hydrogelation that extends beyond conventional salting‐out‐based printing concepts [57].

Due to the above‐established microscopic robustness, hydrogels also resist aggressive chemical environments. In fact, when lowering the pH of a gelled 3 mM solution of **A^o^** to 1, the hydrogel remained stable as long as it was kept at elevated temperature (Figure 5a). Only when the solution was cooled to room temperature, acid‐catalyzed hydrolysis occurred as indicated by a complete loss of color corresponding to the Salphen chromophore and gelation ability. After hydrolysis, heating only led to precipitation, identical to the behavior of stand‐alone PNIPAM. Accordingly, the hydrogel was also protected against transmetallation with Ni(II) even at 90°C (Figure 5b). For comparison, in DMSO, where **A^o^** was not able to assemble, full transmetallation with Ni(II) occurred as indicated by a drastic color change to dark red and confirmed by ^1^H NMR spectroscopy (see Figure S29). This does not only underline the shielding capability of the higher‐order structures, even preserving structural integrity on the macroscopic scale, but also highlights the potential of hierarchical assemblies to selectively control and program chemical reactivity in a stimulus‐responsive manner.

**FIGURE 5:**
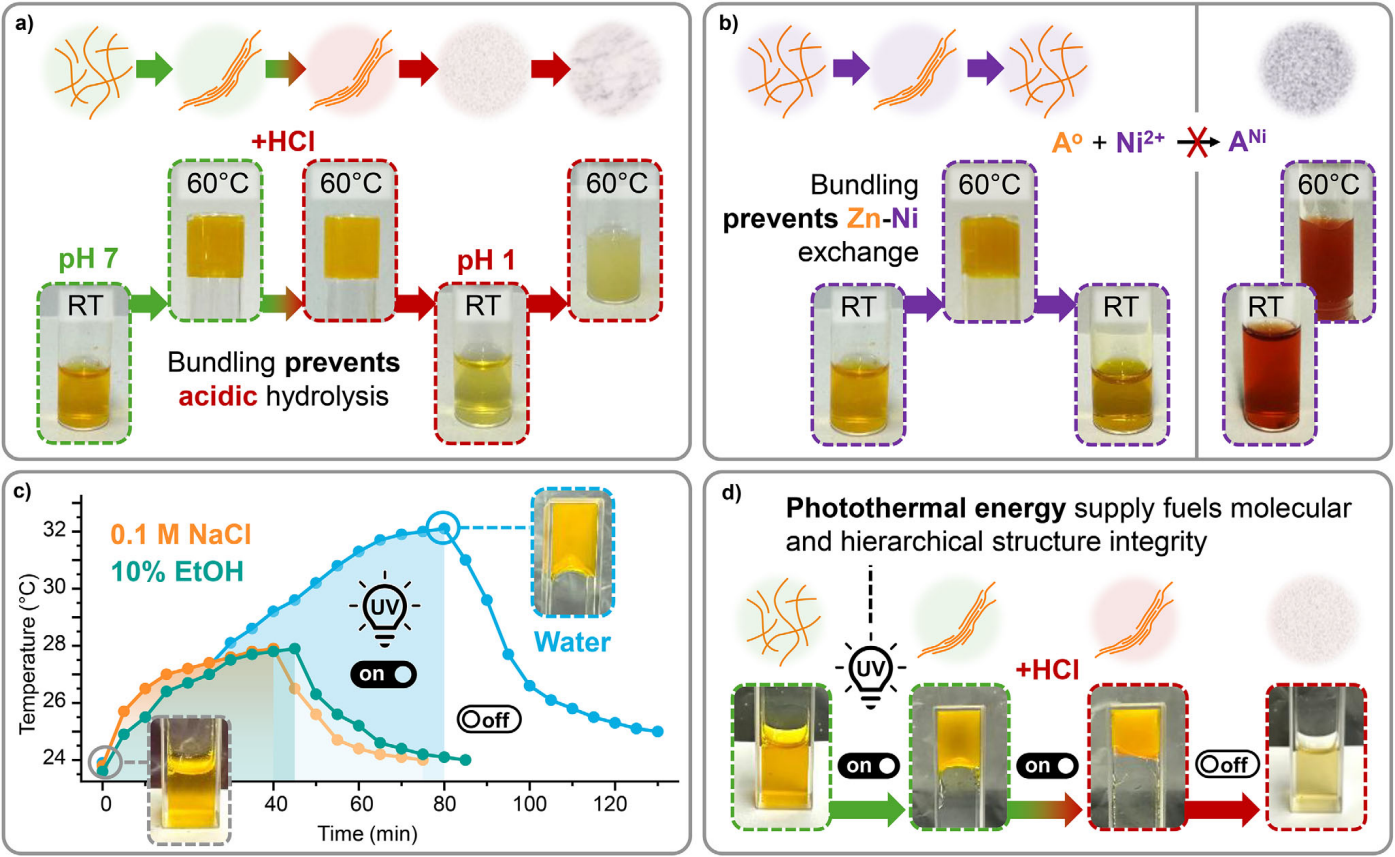
Photographs of a 3 mM solution of **A^o^** and a schematic depiction showcasing how thermally triggered bundling allows for control over (a) acidic hydrolysis and (b) transmetallation with Ni(II). A 3 mM solution of **A^Ni^** is shown for comparative purposes (c) Photothermally triggered gelation by UV irradiation of 3 mM solutions of **A^o^** in water, 0.1 M NaCl and 15 vol% EtOH solution, respectively (370 nm, 40 W, 1.5 mL in 10 mm × 10 mm Quartz cuvette). (d) Photographs of a 3 mM solution of **A^o^** and a schematic depiction of the light‐controlled hydrogelation preventing hydrolysis of the nanofibers.

Up to this point, bundling was exclusively induced under equilibrium conditions. The inherent photophysical properties of the Zn(II) Salphen unit, however, enabled us to exploit light as a controllable energy input, analogous to complex biological processes that operate out of equilibrium and are sustained by a continuous energy supply with light. **A^o^** is a poor emitter with a photoemission lifetime of 0.55 ns at 1 mM meaning that emission occurs from a singlet excited state. As this class of zinc complexes typically exhibits low quantum yields, most excited states decay non‐radiatively, suggesting that most absorbed photon energy is converted into thermal energy [58]. We hypothesized that this property allows the Salphen chromophore to locally generate heat upon irradiation via photothermal heating [59, 60, 61]. Accordingly, we irradiated an aqueous solution of **A^o^** with a UV lamp (370 nm, 40 W) at 15 cm distance and monitored the solution temperature over time (Figure 5c). Indeed, we observed a continuous temperature increase up to a certain plateau value at which the cooling rate from the sample surrounding equals the internal photothermal heating rate. The heating rate increases with increasing concentration of **A^o^**, consistent with a photothermal heating mechanism (see Figure S30). Although the heating rates and plateau temperature of the sample solutions depend on the dimensions of the vessel, in standard UV–vis cuvettes (10 mm × 10 mm) photothermal heating was sufficient to increase the temperature of a 3 mM solution to 32°C, triggering hydrogelation after 80 min of irradiation. After light was switched off, the sample cooled to ambient temperature, causing re‐liquefaction after 50 min. Accordingly, during irradiation, which is a constant energy supply and necessary due to dissipation of thermal energy into the surrounding, the bundling of fibers represents an out‐of‐equilibrium state as cessation of the photoenergy supply disentangles the bundled strands. The temperature required for gelation point can be significantly reduced by increasing ionic strength or changing solvent composition. This leads to shorter photothermal heating durations before bundling occurs. Indeed, solutions of **A^o^** in 0.1 M NaCl or 10 % EtOH, under otherwise identical conditions, gel at 27°C or 28°C, respectively. Accordingly, photothermally activated hydrogelation is already achieved after 40‐45 min, thus reducing the gelation time by a factor of two (Figure 5c). As the photo‐induced bundling does not rely on chemical rearrangements avoiding potential side reactions, it is fatigue resistant. After turning off the UV lamp and cooling, irradiation cleanly regenerates the hydrogel.

Finally, we introduced acidity as a representative chemically destructive stimulus, which could be mitigated only when light acted as a temporal energy source (Figure 5d). Irradiating a pH‐neutral aqueous solution of **A^o^** until hydrogelation occurred and then lowering the pH to below 1 created an out‐of‐equilibrium state in which irradiation not only maintained the integrity of the bundles but also of the individual monomers themselves. When the UV lamp was switched off, the energy supply ceased, causing the sample to cool, the bundles to release the nanofibers, and their monomeric units to be hydrolyzed. Hence, irradiation in the hierarchically structured state ensured survival of the simplest assembly unit, the Salphen complex itself, in the hostile medium. This provided an instructive analogy to protective mechanisms in nature, where acid‐resistant bacteria actively expend energy to maintain intracellular pH homeostasis under proton‐rich conditions [62]. To achieve this, these organisms deploy a suite of bacterial acid stress responses—including membrane lipid remodeling, proton pumps, and regulated stress‐response pathways—to withstand harmful acidic conditions [63]. Here, we have demonstrated an analogous concept for an entirely synthetic system.

## 3 Conclusion

In this work, we introduced PNIPAM‐functionalized Zn(II) Salphen nanofibers that spontaneously form in dilute aqueous solution. By applying a set of external stimuli (temperature, ionic strength, and solvent composition), we induced fully reversible higher‐order aggregation, as directly visualized by cryo‐TEM. This hierarchical structuring markedly enhanced stability and modulated the intrinsic photophysical properties of the assemblies. Bundling and protection occurred selectively in mixtures of fibers. At the macroscopic level, higher‐order aggregation gave rise to reversible hydrogelation at unusually low concentrations and under external control, allowing tuning of the gelation temperature and gel shape. Moreover, UV light irradiation triggered photothermal bundling, generating a hierarchically structured state that protects the smallest assembly unit and only persists while energy is supplied.

Collectively, these results demonstrate that controlled hierarchical self‐assembly across multiple length scales enables programmable protection of supramolecular Zn(II) Salphen assemblies in various environments. Conceptually, this behavior parallels biological systems, in which hierarchical organization and energy‐driven processes underpin robustness, compartmentalization, and adaptive function. The resulting multiscale cascade—from molecular design and nanoscopic self‐assembly to microscopic bundling and macroscopic hydrogelation—integrates multi‐stimuli responsiveness with programmable reactivity across all dimensions. Overall, this platform provides a modular strategy for designing dynamic, stimuli‐responsive soft materials with relevance to, for example, nano‐ and biotechnology, optoelectronics, and sensing.

## Author Contributions

**Merlin R. Stühler**: conceptualization, data curation, investigation, writing – original draft, writing – review and editing, formal analysis. **Rainer Haag**: funding acquisition, supervision, writing – review, and editing. **László Mérai**: methodology, data curation, investigation, formal analysis, writing – review and editing. **Quinn A. Besford**: funding acquisition, supervision, writing – review and editing. **Kai Ludwig**: investigation, formal analysis, supervision. **Alex J. Plajer**: conceptualization, supervision, funding acquisition, project administration, writing – original draft, writing – review and editing.

## Conflicts of Interest

The authors declare no conflicts of interest.

## Supporting information




**Supporting File**: adma74151‐sup‐0001‐SuppMat.pdf.

## Data Availability

The data that supports the findings of this study are available in the supplementary material of this article
