# Supplementary material for: Stimuli‐Responsive Hierarchical Structuring Controls Survival and Programs Hydrogelation of Supramolecular Metallofibers
Source: Adv Mater. 2026 Jul 16;38(56):e74151. doi: 10.1002/adma.74151 (PMC13648901; doi:10.1002/adma.74151)
Supplement: Supplementary file 1 [file ADMA-38-e74151-s001.pdf]

Supporting Information for

Stimuli-Responsive Hierarchical Structuring Controls Survival and Programs Hydrogelation of Supramolecular Metallofibers

Merlin R. Stühler,[a,b] László Mérai,[c] Kai Ludwig,[b] Rainer Haag,[b] Quinn A. Besford,[c] Alex J. Plajer[a,d]*

[a] Makromolekulare Chemie, Universität Bayreuth, Universitätsstraße 30, 95447 Bayreuth; [b] Institut für Chemie und Biochemie, Freie Universität Berlin, Takustraße 3, 14195 Berlin; [c] Leibniz-Institut für Polymerforschung e.V., Hohe Straße 6, 01069 Dresden; [d] Bayrisches Polymer Institut (BPI), Universität Bayreuth, Universitätsstraße 30, 95447 Bayreuth. *email: alex.plajer@uni-bayreuth.de

Table of Contents

S01 Methods 2
S02 Experimental 4
S03 Cryogenic Transmission Electron Microscopy/ Cryogenic Electron Tomography 10
S04 Fluorescence Lifetime Imaging Microscopy 16
S05 Rheology 18
S06 Transmetallation with Ni(II) 20
S07 Photothermally Induced Hydrogelation Experiments 21
S08 References 21

S01 Methods

Solvents and reagents were obtained from commercial sources and used as received unless stated otherwise. Carboxylic acid precursors and mPEG-functionalized monomers (**B°**) were synthesized after literature protocolls.[1]

Cryogenic transmission electron microscopy (cryo-TEM): 3.7 µL of the sample were applied to plasma treated R1.2/1.3 holey carbon grids (Quantifoil Microtools, Großlöbichau, Germany), blotted for 3 s and vitrified in liquid ethane using Vitrobot Mark IV (FEI Company, Eindhoven, Netherlands) set to 95% humidity. The waiting time was adjusted according to temperature: grids blotted at 10 °C and those containing EtOH (to avoid extensive evaporation) had a waiting time of 3 s, whereas grids blotted at higher temperatures (e.g., 36 °C) had a waiting time of 30 s to ensure equilibration of the sample solution. Imaging was conducted manually on a FEI Talos Arctica 200 kV TEM, equipped with a Falcon3 EC direct electron detector using Low dose mode in TIA (FEI company, Eindhoven, Netherlands)

Cryo-electron tomography (cryo-ET): To obtain spatial information, tomograms were recorded on the TALOS® ARCTICA transmission electron microscope (ThermoFisher Scientific Inc., Waltham (MA), USA) at 200 kV. For this purpose, single axis tilt series (±64° in 2° tilt angle increments) were acquired with a FEI Falcon 3EC 4k×4k direct electron detector at 17500 K primary magnification with a total dose lower than 120 $e^-/Å^2$. Reconstruction of the tomograms was performed with binned data (binning factor 2) using ThermoFisher Inspect3D software, version 3.1.0. The increased contrast provided by the phase plate facilitated the alignment of the individual tilt images, but also slightly reduced the transmission of high spatial frequencies. The 3D voltex presentation was prepared using Amira, Version 6.2 (ThermoFisher Scientific Inc., Waltham (MA), USA).

Dynamic light scattering (DLS) data were obtained using a Malvern Zetasizer Ultra equipped with a 10 mW He-Ne laser operating at a wavelength of 632.8 nm, and the analysis was conducted using the ZS Xplorer software. All data points are an average result of three individual size measurements.

Electrospray ionization (ESI) mass spectra were obtained using a Waters UPLC-Synapt G2-S HDMS. The sample solutions were introduced into the mass spectrometer using a syringe pump at a flow rate of 10 µL/min. Each measurement was averaged over 1 minute to account for instabilities in the signal.

Fluorescence Lifetime Imaging Microscopy (FLIM) and time correlated single photon counting (TCSPC) data were measured with an inverted confocal scanning microscope (MT200, PicoQuant) incorporating a 100×air objective (UPFLN, NA 0.9, Olympus, Japan). As a measurement cell, a confined stainless-steel sample holder was used, with a precleaned (sonication in abs. EtOH), transparent glass cover slide as a bottom side. During the lifetime imaging measurements, the ~200 µL samples were transferred to the measurement cell, then they were subjected to excitation using a laser with an emission wavelength of λ = 405 nm (LDH405) and a 10.00 MHz repetition rate at room temperature (21 °C). The signal was guided through a long pass filter with a cutoff below 425 nm and detected with a SPCM AQRH single photon counting module (Excelitas). The scanning rate was 2 ms/pixel. The intensity-average fluorescence lifetimes ($\tau_{int.avg.}$) were obtained as a result of 3- or 4-parameter exponential tail-fitting on the obtained global (cumulative for the whole image) decay curves (intensity vs. time). The highest time value of the fitting range was 24.5 ns, while the lowest values were arbitrarily

selected (in the range of ~1.9-2.5 ns) with excluding the instrument response, achieving a relatively high fitting accuracy and avoiding the exclusion of short-lifetime emission components. At each sample composition, at least 3 images were recorded, and the obtained average $\tau_{int.avg}$. values were plotted as a function of solvent composition.

Nuclear magnetic resonance (NMR) spectra were recorded using a JEOL JNM-ECA 400II and JOEL ECZ600 spectrometer. Chemical shifts are referenced to the residual resonance of the deuterated solvents.

Photoemission spectra were recorded on FP-6500 spectrofluorometer (Jasco, 4-21, Sennin-cho 2-chome, Hachioji, Tokyo 193-0835, Japan). All measurements were performed in 10 mm quartz cuvettes (Hellma GmbH & Co. KG, Klosterrunsstraße 5, D-79379 Müllheim) at the specified temperature using a concentration of 100 µM.

Rheology measurements were performed using a Kinexus rheometer (NETZSCH GmbH, Selb, Germany) equipped with a coned plate of 20 mm in diameter and a 1° angle. For variable temperature rheology measurements, 70 µL of the specified solution were added onto the stage at room temperature and additional solvent was placed around the sample, without getting in contact with it, to ensure a saturated atmosphere and prevent the sample from drying out upon heating. Under a closed hood, the sample was equilibrated to the starting temperature for 5 minutes before the measurement started. All temperature-dependent measurements were performed using a frequency of 1 Hz, a shear strain of 1 % and a ramp speed of 2 °C/min.

UV-Vis absorption spectra were measured on a Varian Cary 8554 UV-Vis spectrophotometer (Agilent Technologies Inc., Santa Clara, USA). All measurements were performed in 10 mm Quartz cuvettes (Hellma GmbH & Co. KG, Klosterrunsstraße 5, D-79379 Müllheim) at the specified temperature using a concentration of 100 µM.

UV-irradiation experiments were conducted using a 370 nm, 40 W KESSIL PR160L-370 Gen 2 UV lamp. 1.5 mL of the respective solution in a Quartz cuvette (Hellma) was irradiated from 15 cm distance for the specified time. The temperature was monitored using a IKA ETS-D5 thermometer.

S02 Experimental

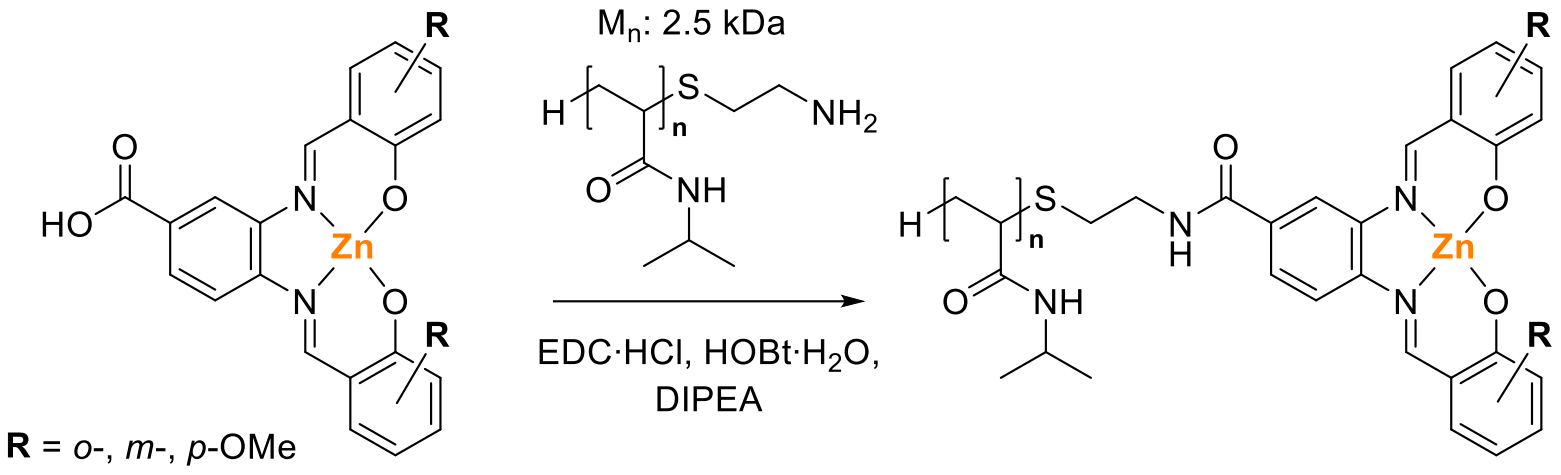

Scheme S1: General synthesis for amide-coupled Salphen complexes.

General procedure for the synthesis of amide-coupled Salphen complexes based on a modified literature method.[2] Under an argon atmosphere, the respective carboxylic acid precursor (1 equiv.) was dissolved in DMSO. Then, 1-ethyl-3-(3-dimethylaminopropyl)carbodiimide hydrochloride (EDC·HCl, 1.5 equiv.), hydroxy benzotriazole hydrate (HOBt·H_2O, 1.5 equiv.) and *N*,*N*-Diisopropylethylamine (DIPEA, 1.5 equiv.) were added, and the mixture was stirred at room temperature typically resulting in a color change from orange to red. After 45-60 minutes, the respective amine functionalized poly(NIPAM) (1 eq.) was added to the reaction mixture and the solution was stirred for 24 h. The crude reaction mixture was transferred into a dialysis tube (1 kDa) and dialyzed against distilled water for 3 days while regularly changing the solvent. The dialysed solution was lyophilized to obtain the desired product as a yellow solid.

A°: ESI-MS *m/z* = [**A°**+K]$^+$ calculated (n=20): 2842.7133; found (n=20): 2842.5143. Yield: 69 %.

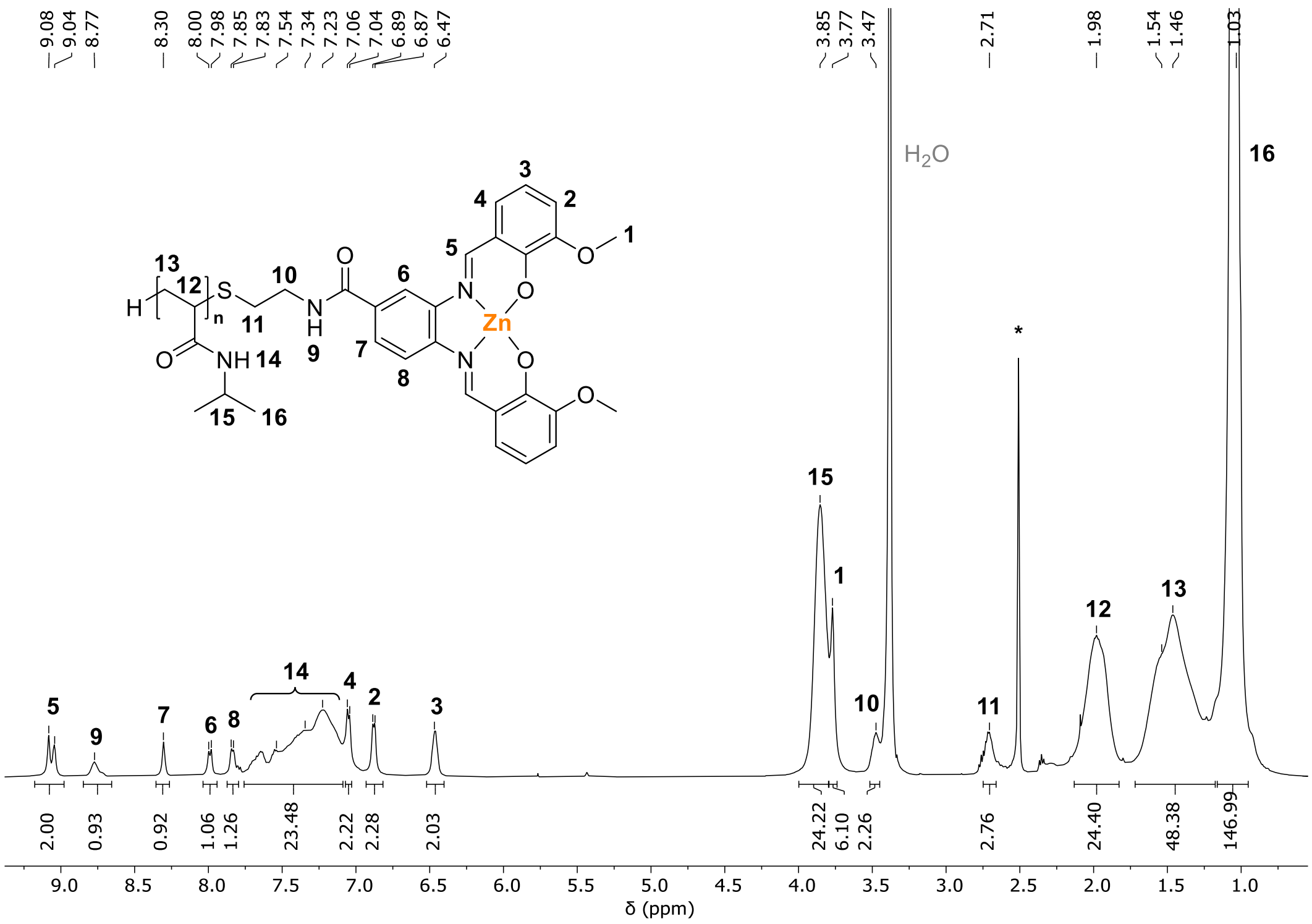

Figure S1: ^1H NMR spectrum (600 MHz, DMSO-d_6) of **A°**. *Denotes solvent.

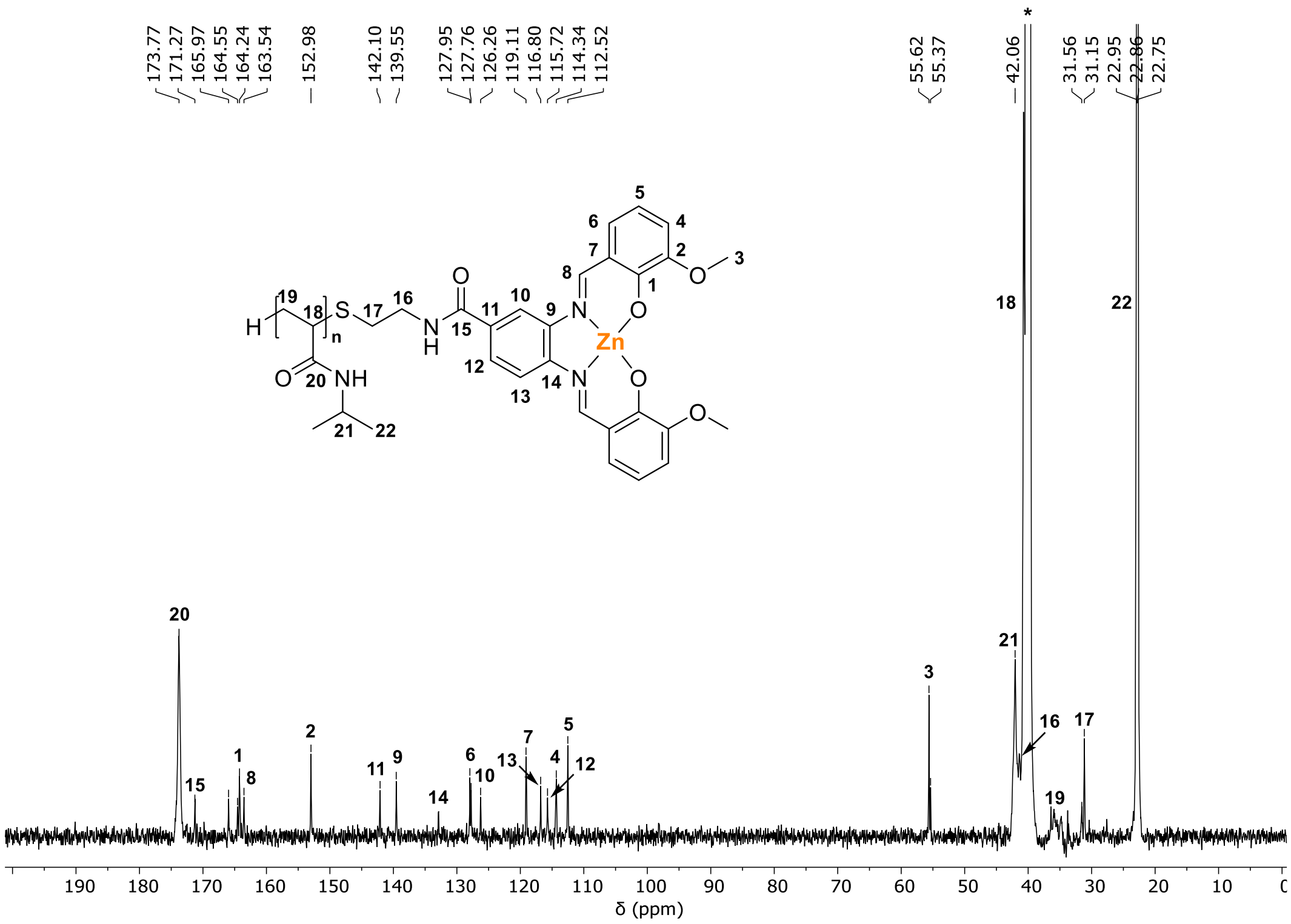

Figure S2: ^{13}C NMR spectrum (151 MHz, DMSO-d_6) of **A°**. *Denotes solvent.

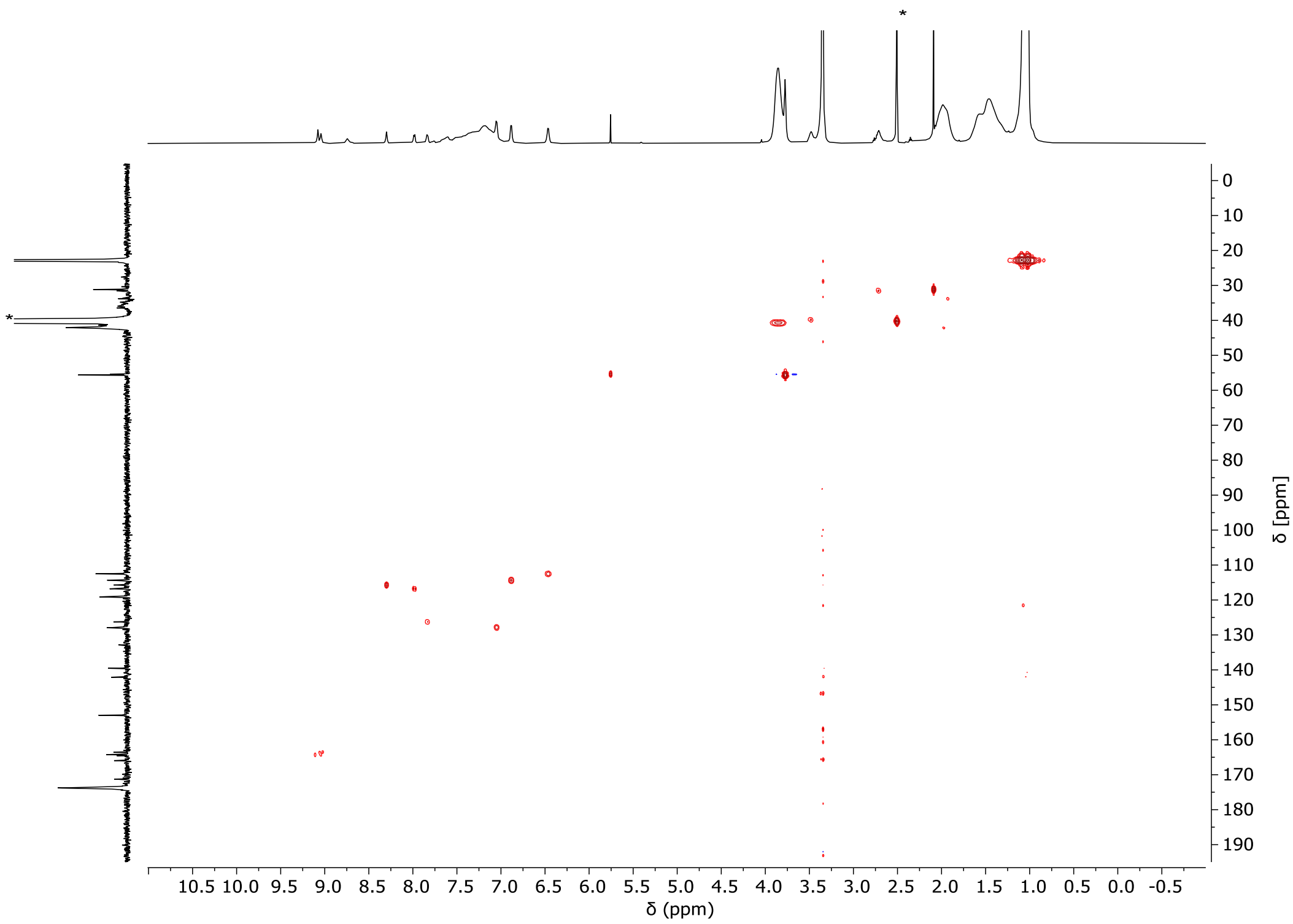

Figure S3: ^{1}H-^{13}C HSQC NMR spectrum (DMSO-d_6) of **A°**. *Denotes solvent.

A^m: ESI-MS m/z = [**A^m**+K]$^+$ calculated (n=20): 2842.7133; found (n=20): 2842.7718. Yield: 89 %.

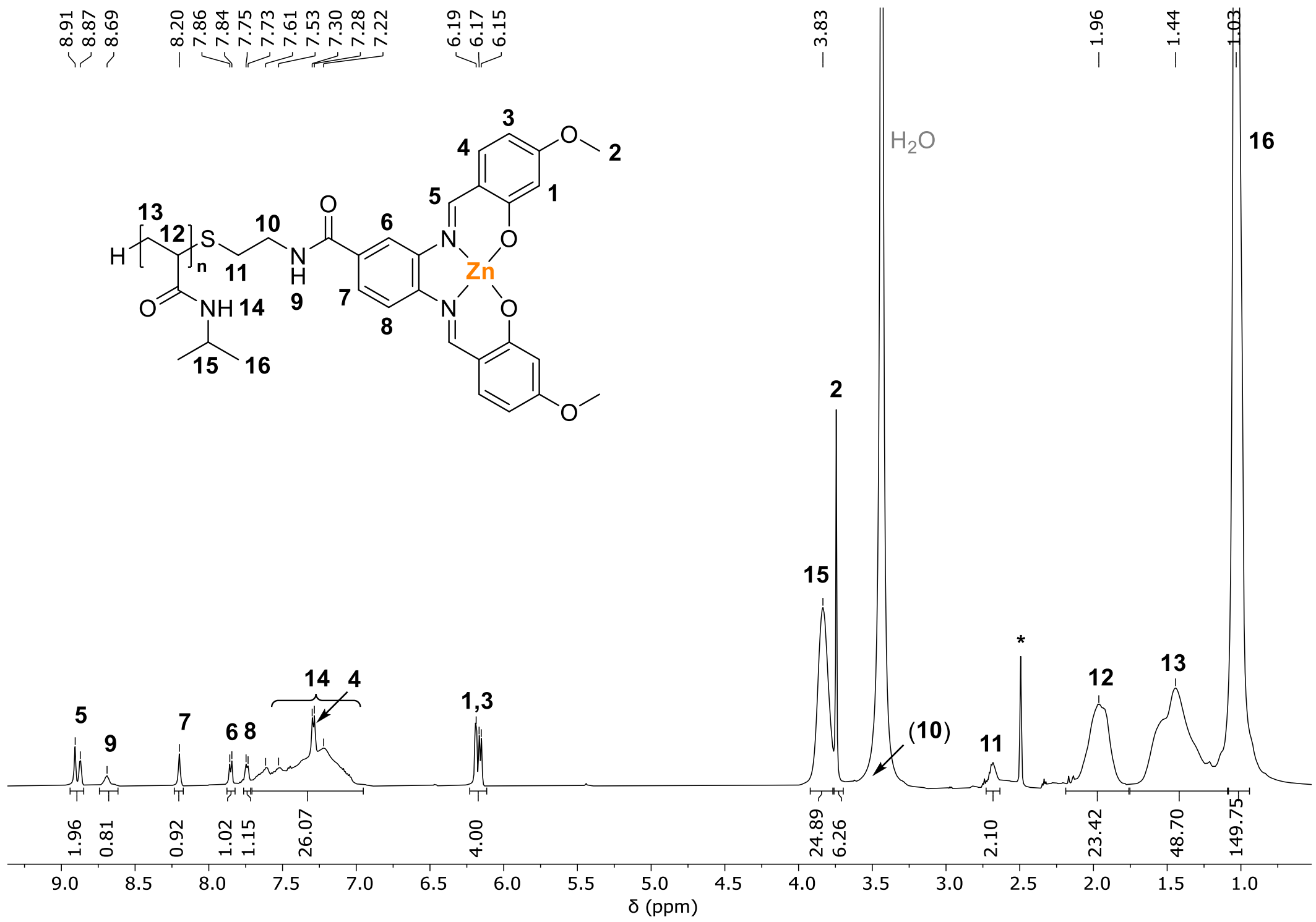

Figure S4: ^{1}H NMR spectrum (600 MHz, DMSO-d_6) of **A^m**. *Denotes solvent.

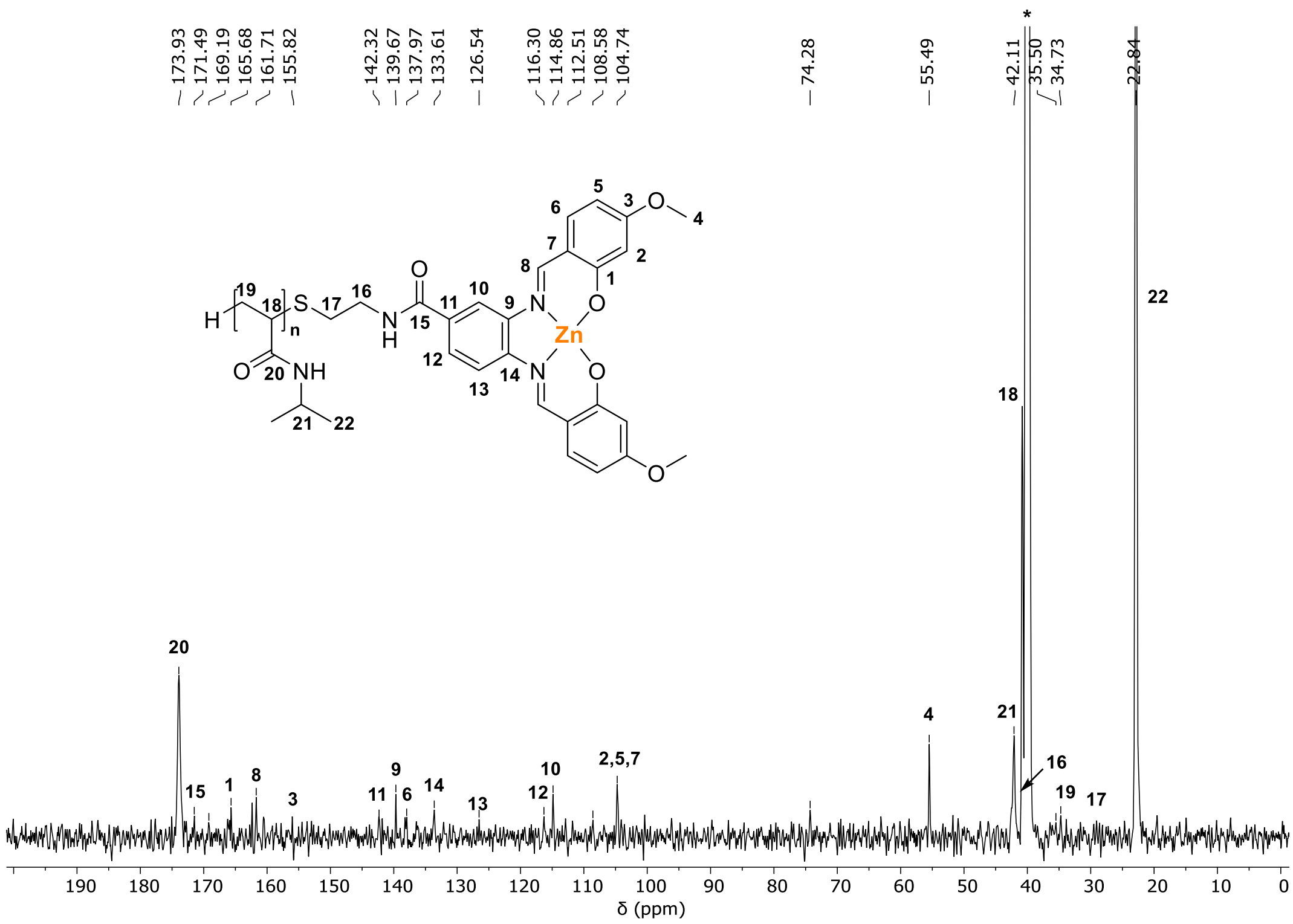

Figure S5: ^{13}C NMR spectrum (151 MHz, DMSO-d_6) of **A^m**. *Denotes solvent.

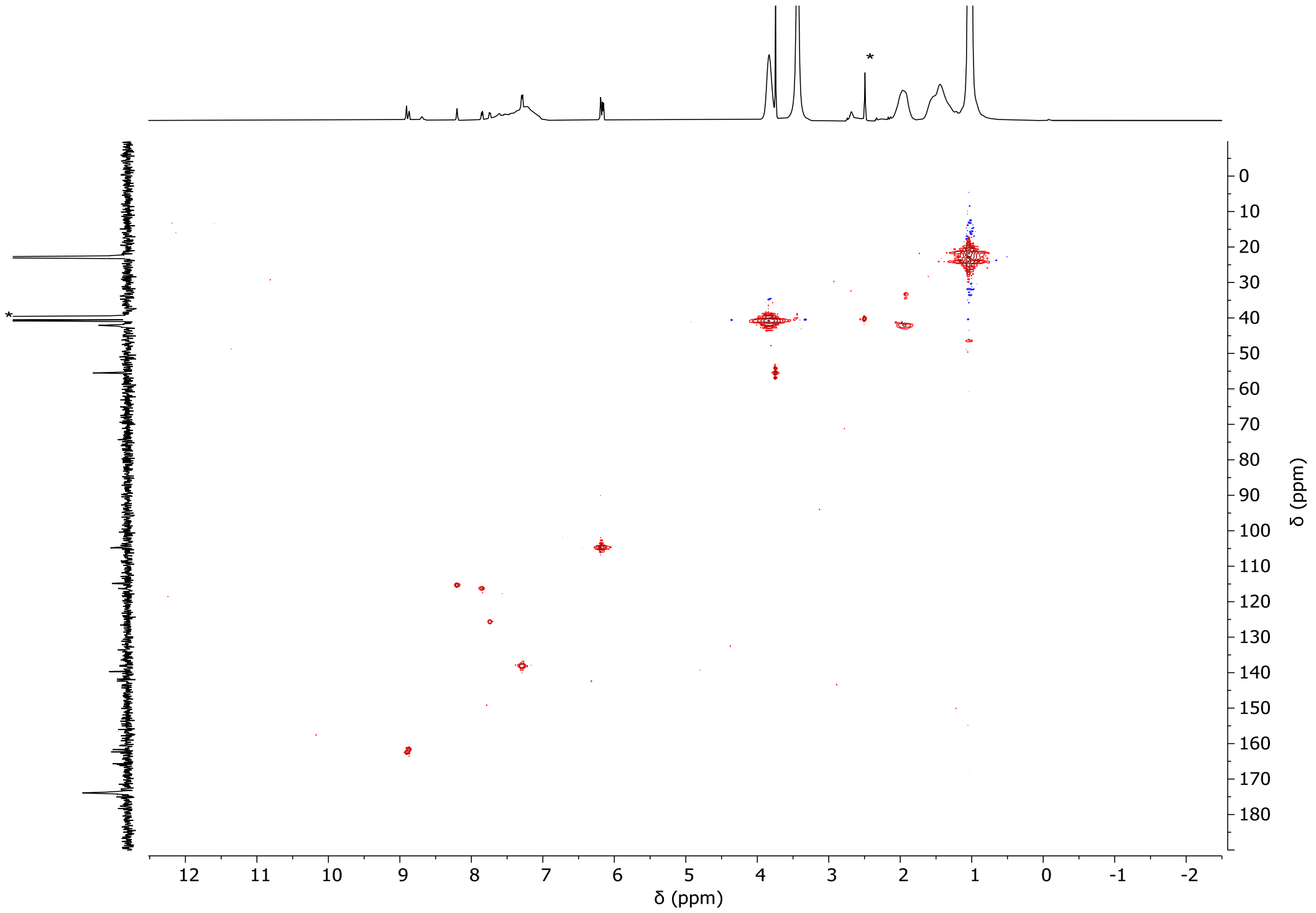

Figure S6: ^1H-^{13}C HSQC NMR spectrum (DMSO-d_6) of **A**^{m}. *Denotes solvent.

A^p: ESI-MS *m/z* = [**A**^{p}+K]$^+$ calculated (n=20): 2842.7133; found (n=20): 2842.7734. Yield: 82 %.

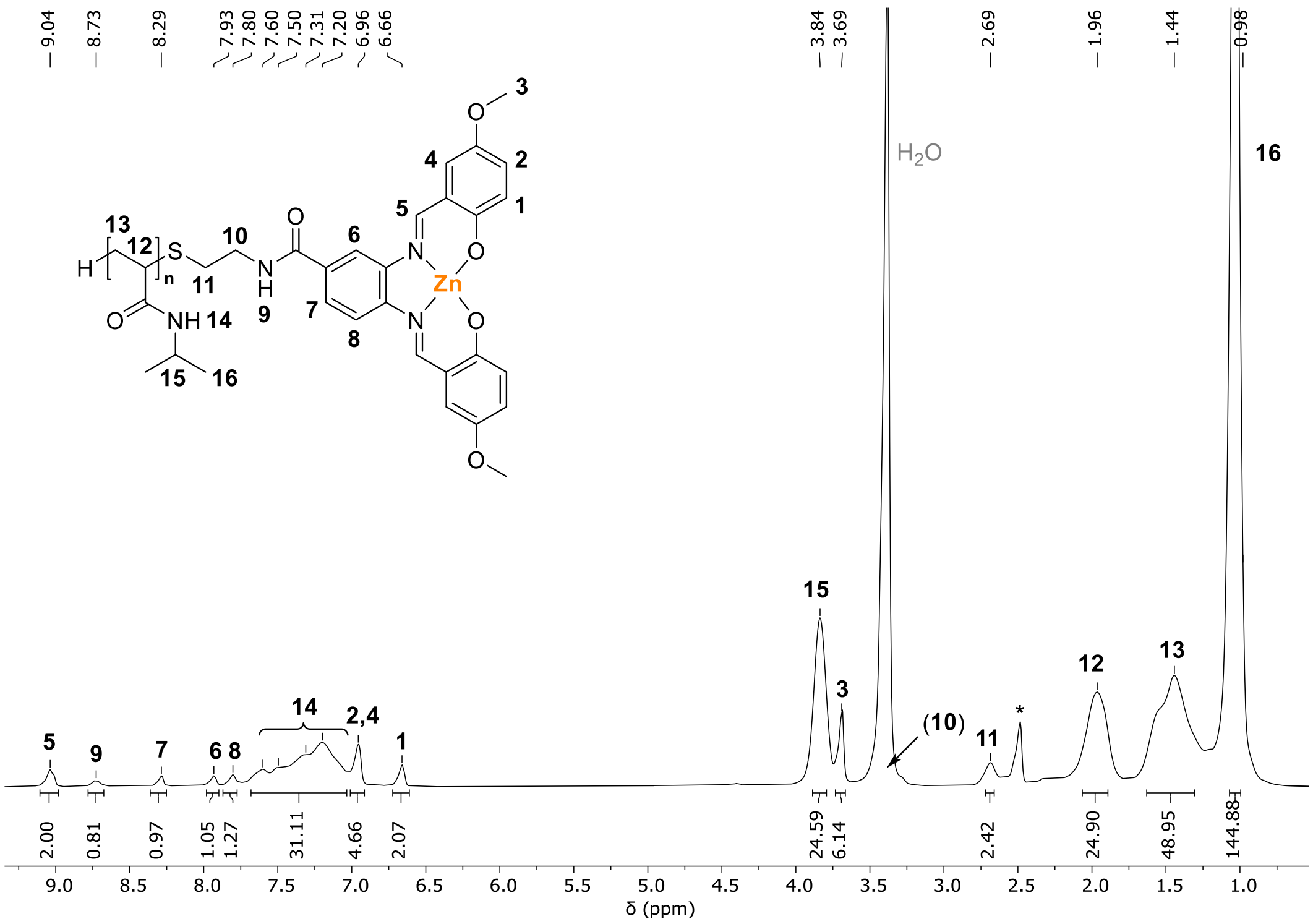

Figure S7: ^1H NMR spectrum (600 MHz, DMSO-d_6) of **A**^{p}. *Denotes solvent.

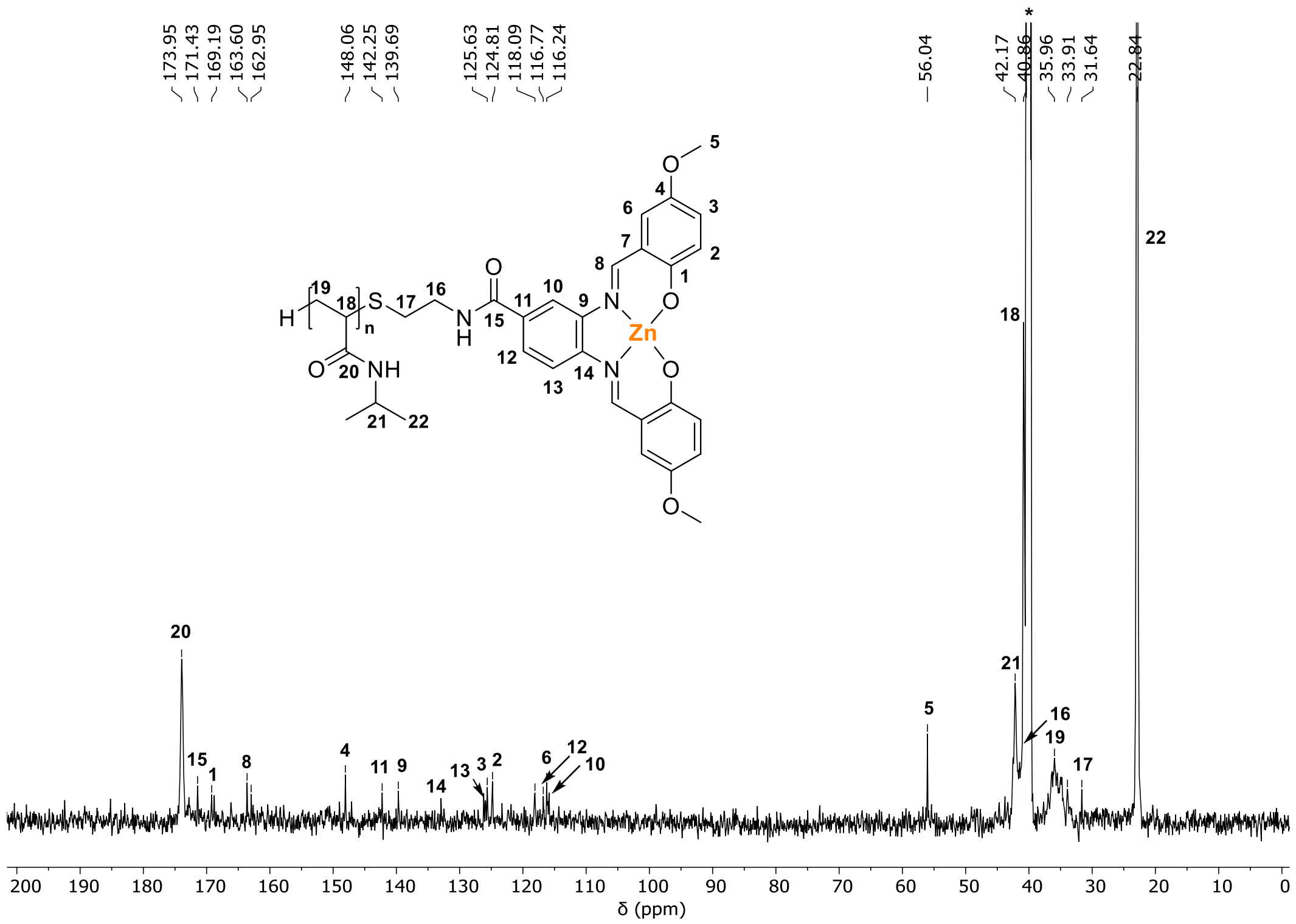

Figure S8: ^{13}C NMR spectrum (151 MHz, DMSO-d_6) of **A^p**. *Denotes solvent.

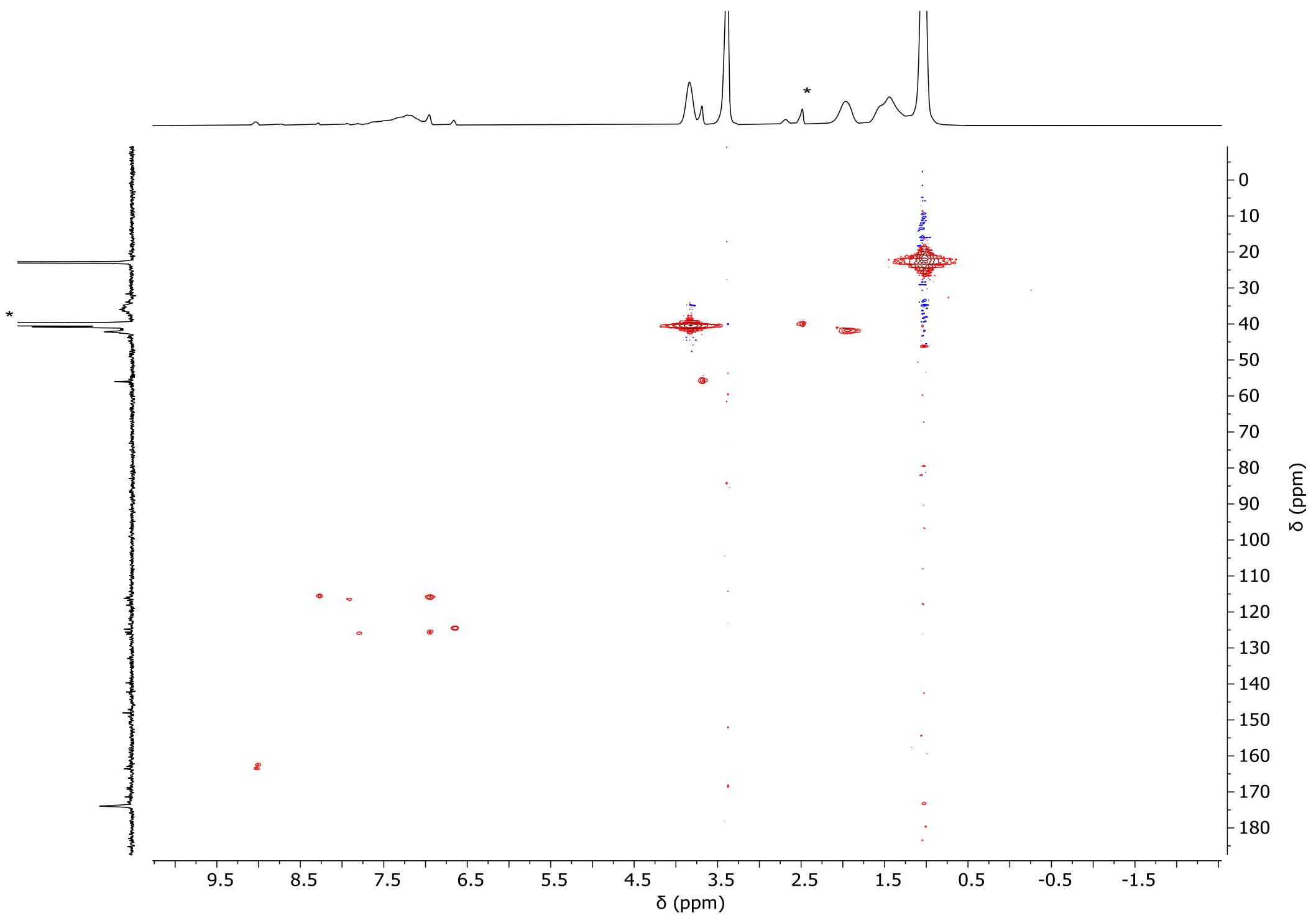

Figure S9: ^{1}H-^{13}C HSQC NMR spectrum (DMSO-d_6) of **A^p**. *Denotes solvent.

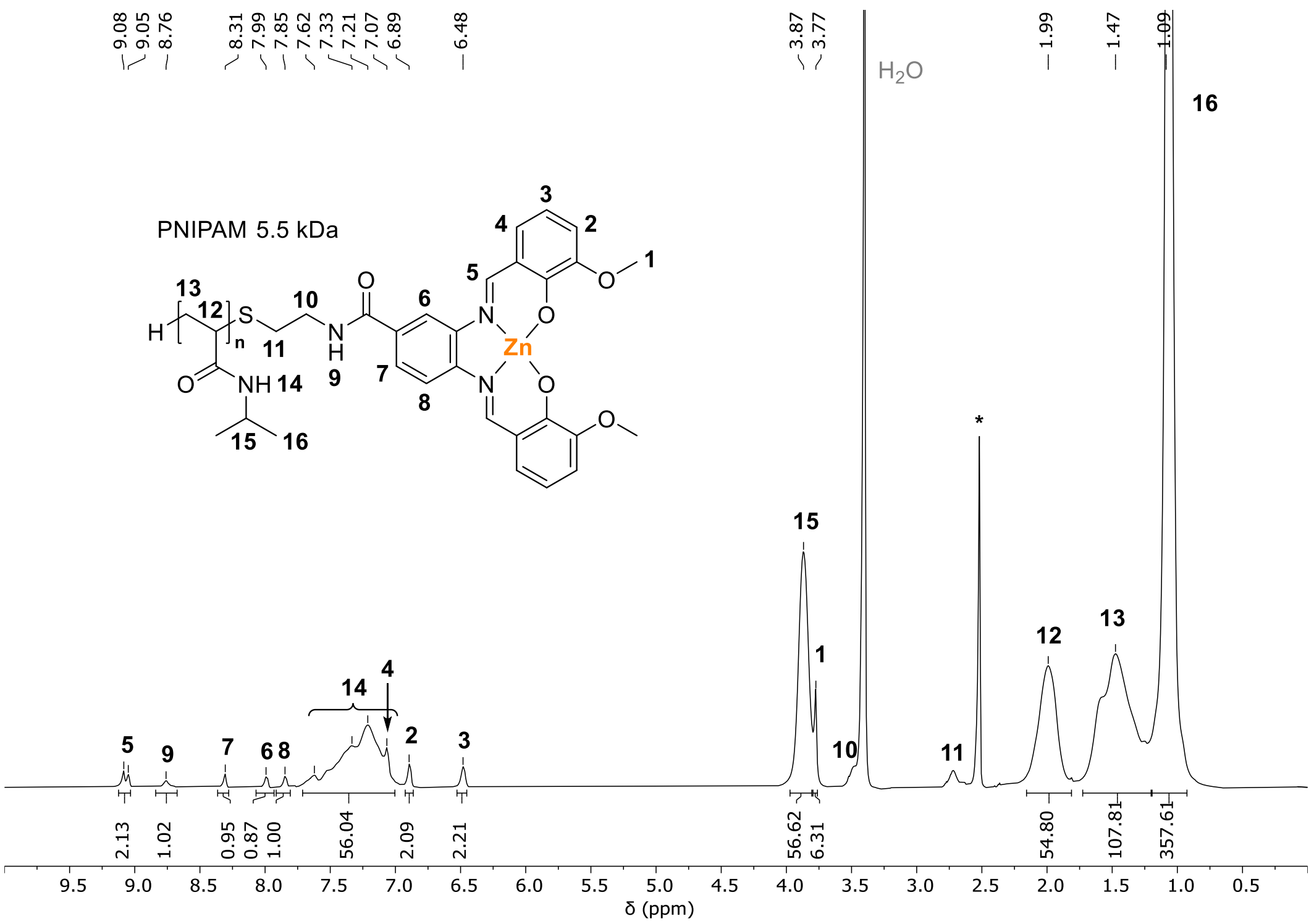

Figure S 10: ^{1}H NMR spectrum (600 MHz, DMSO-*d₆*) of 5.5 kDa PNIPAM-**A°**. *Denotes solvent.

S03 Cryogenic Transmission Electron Microscopy/ Cryogenic Electron Tomography

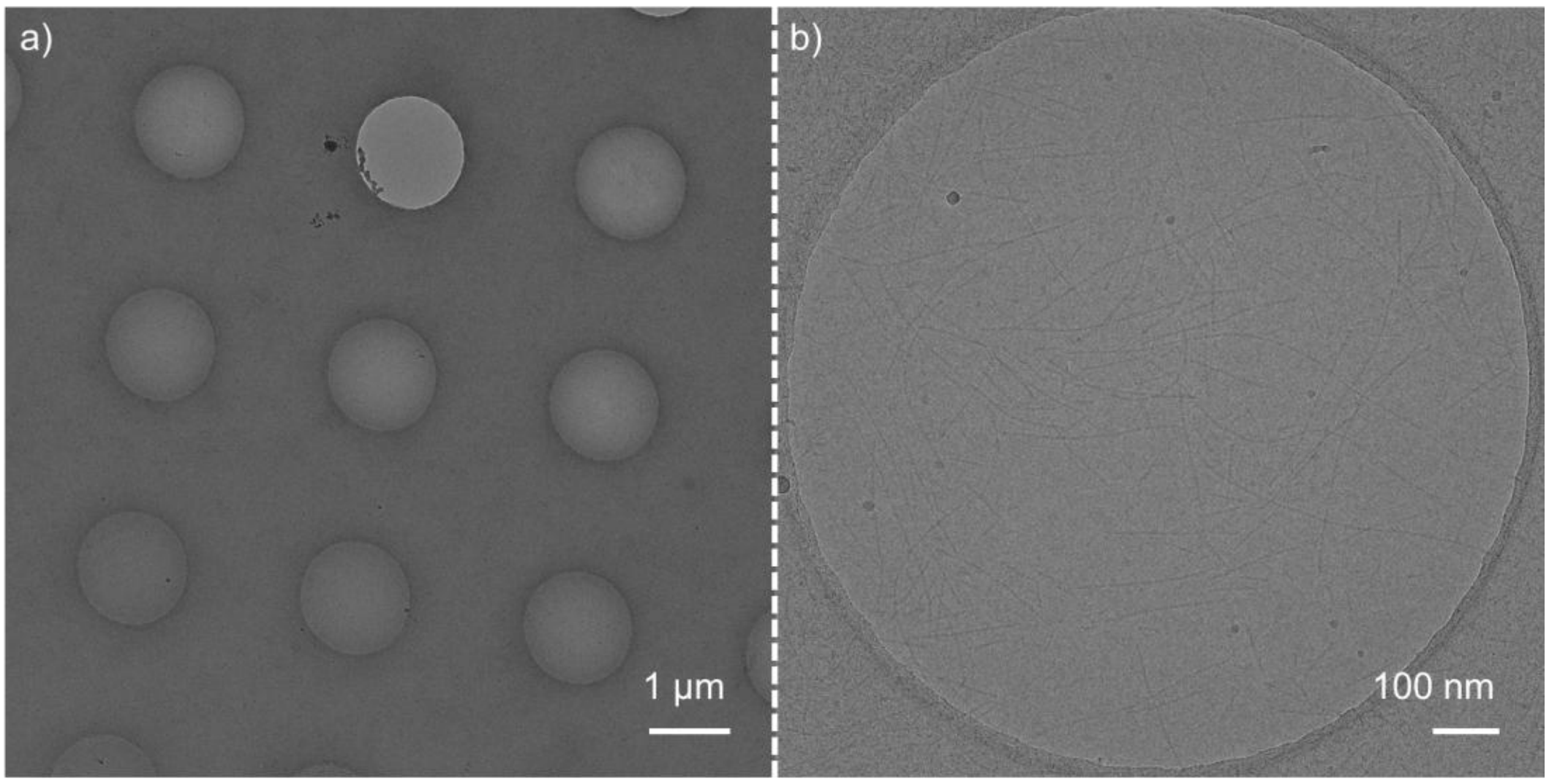

Figure S11: Cryo-TEM images at a) 3400x and b) 28000x magnification of **A°** in water vitrified at 10 °C.

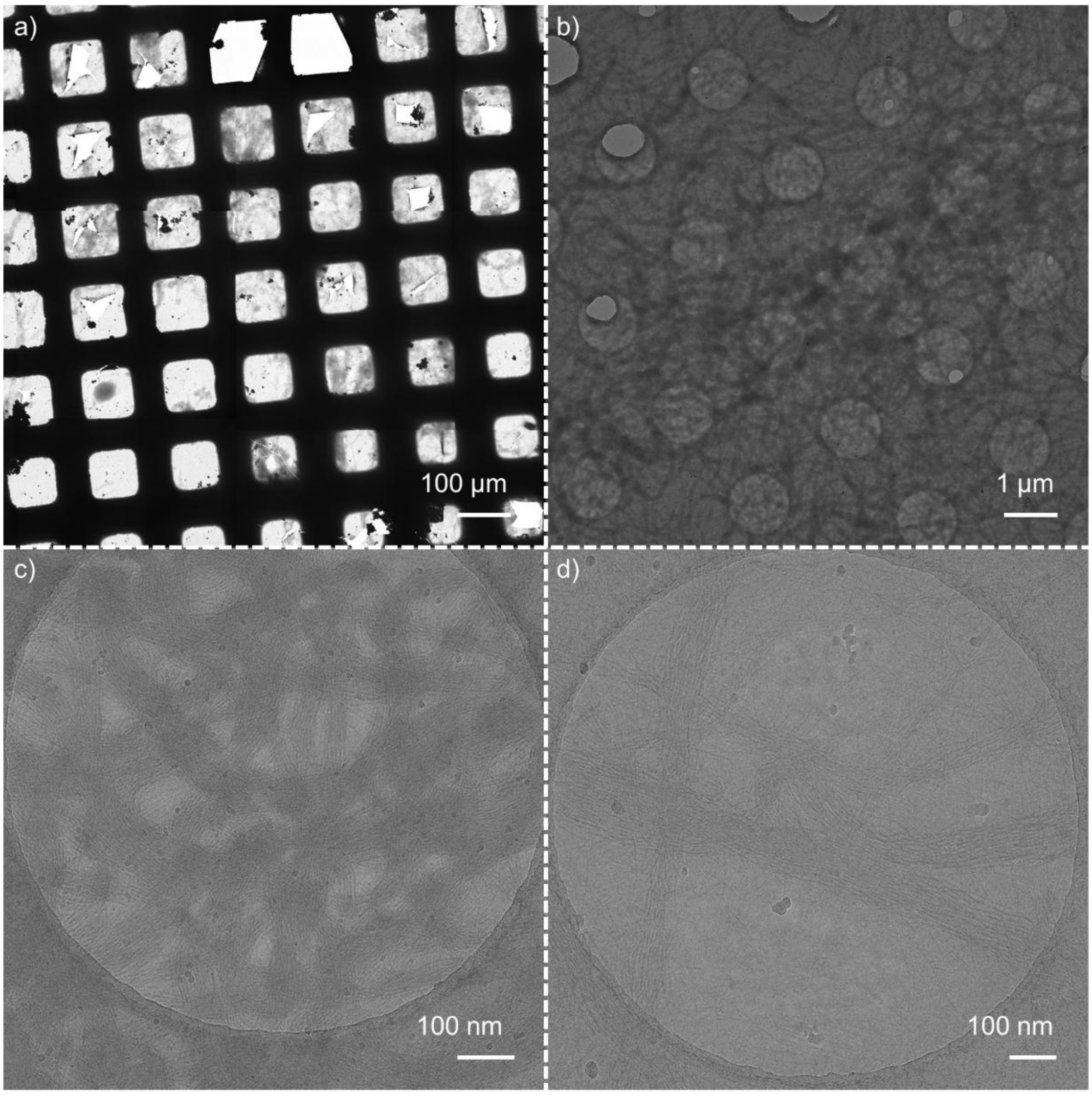

Figure S12: Cryo-TEM a) atlas and images at b) 3400x and c-d) 28000x magnification of **A°** in water vitrified at 36 °C.

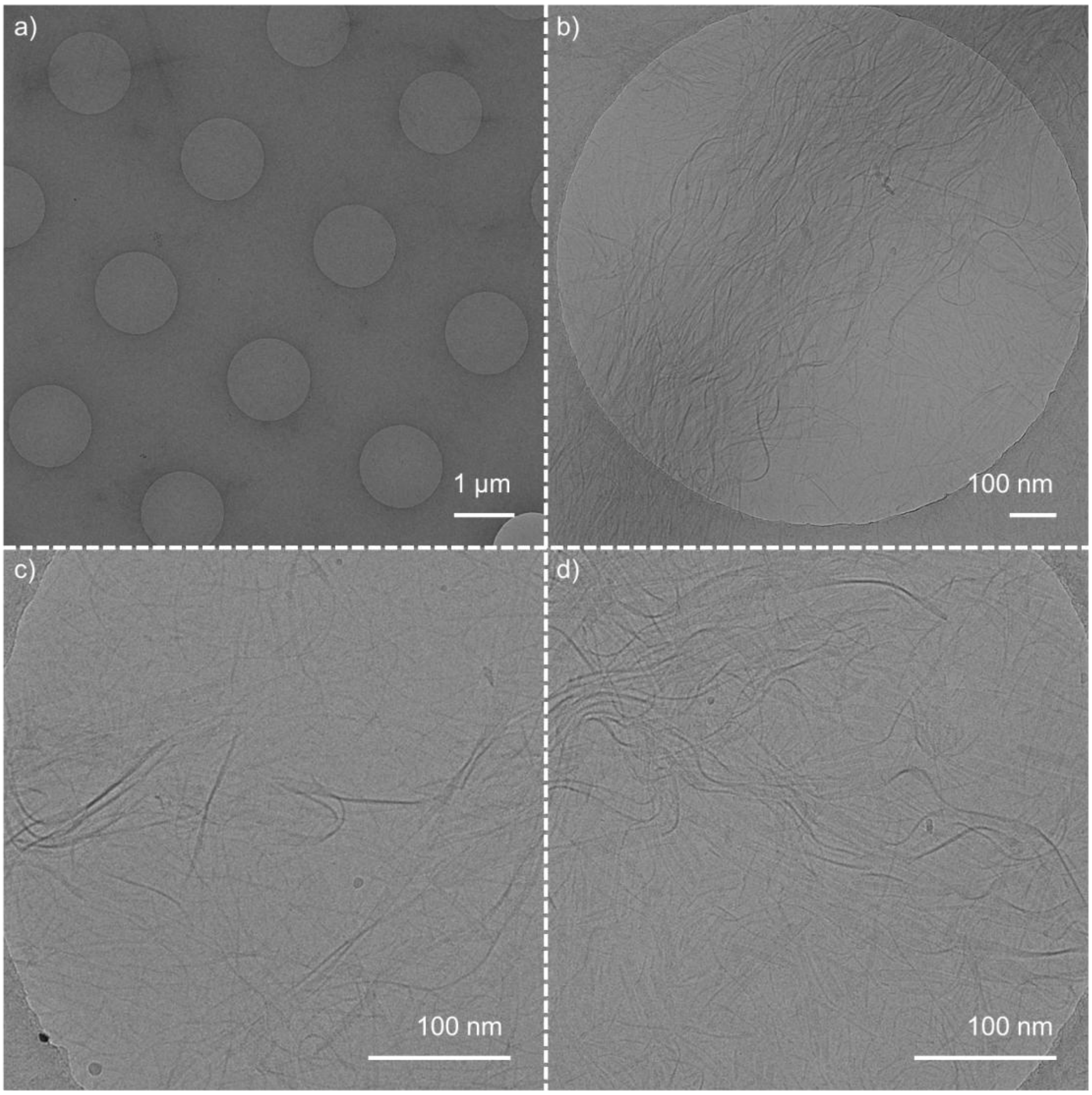

Figure S13: Cryo-TEM images at a) 3400x and b-d) 28000x magnification of $\mathbf{A^m}$ in water vitrified at 10 °C.

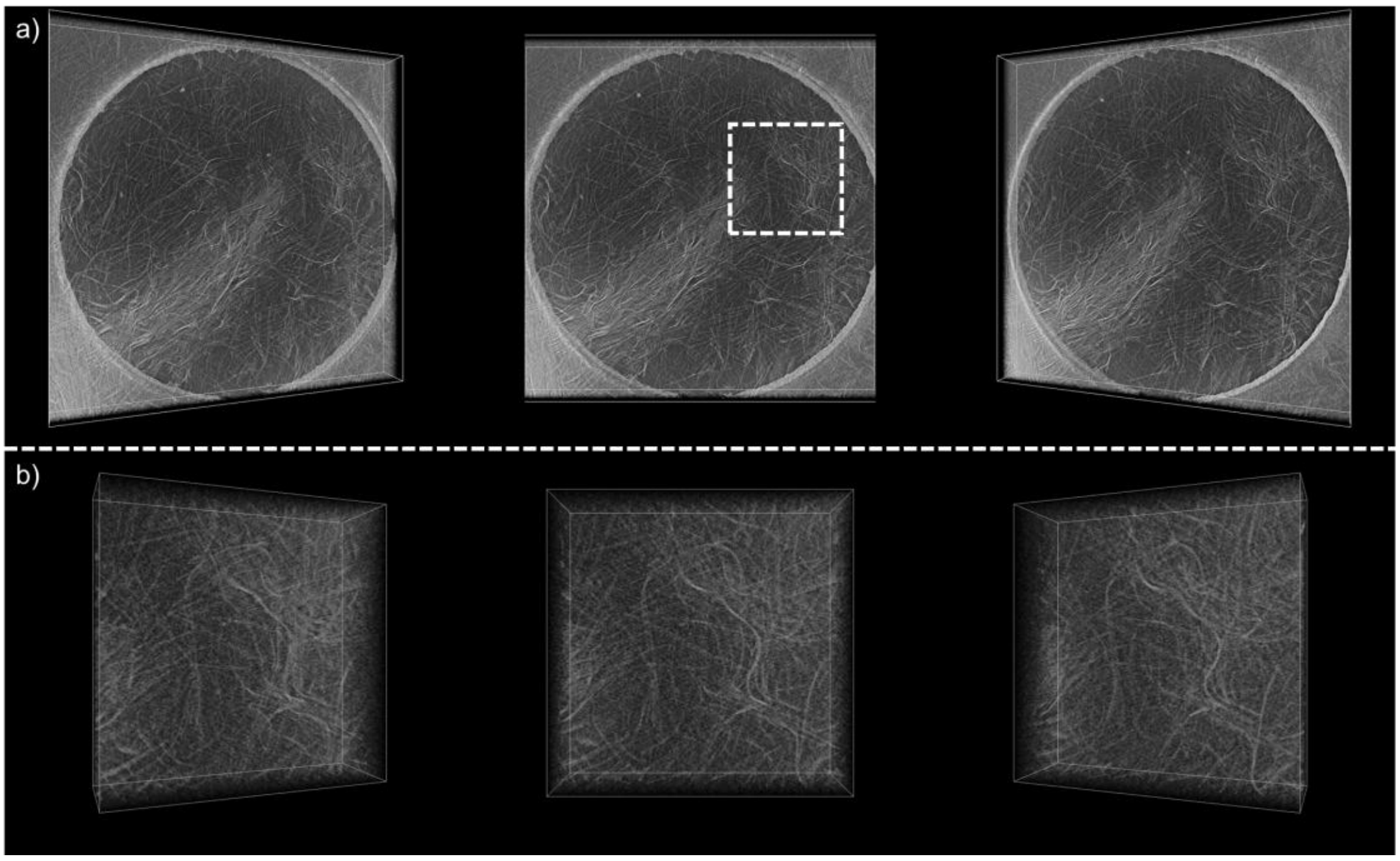

Figure S14: Cryo-ET Voltex representation (inverted contrast) of the 3D volume, calculated from a cryo-TEM tilt series of +/-64° (increment of 2°), of a 0.1 mM solution of $\mathbf{A^m}$ in water vitrified at 10 °C.

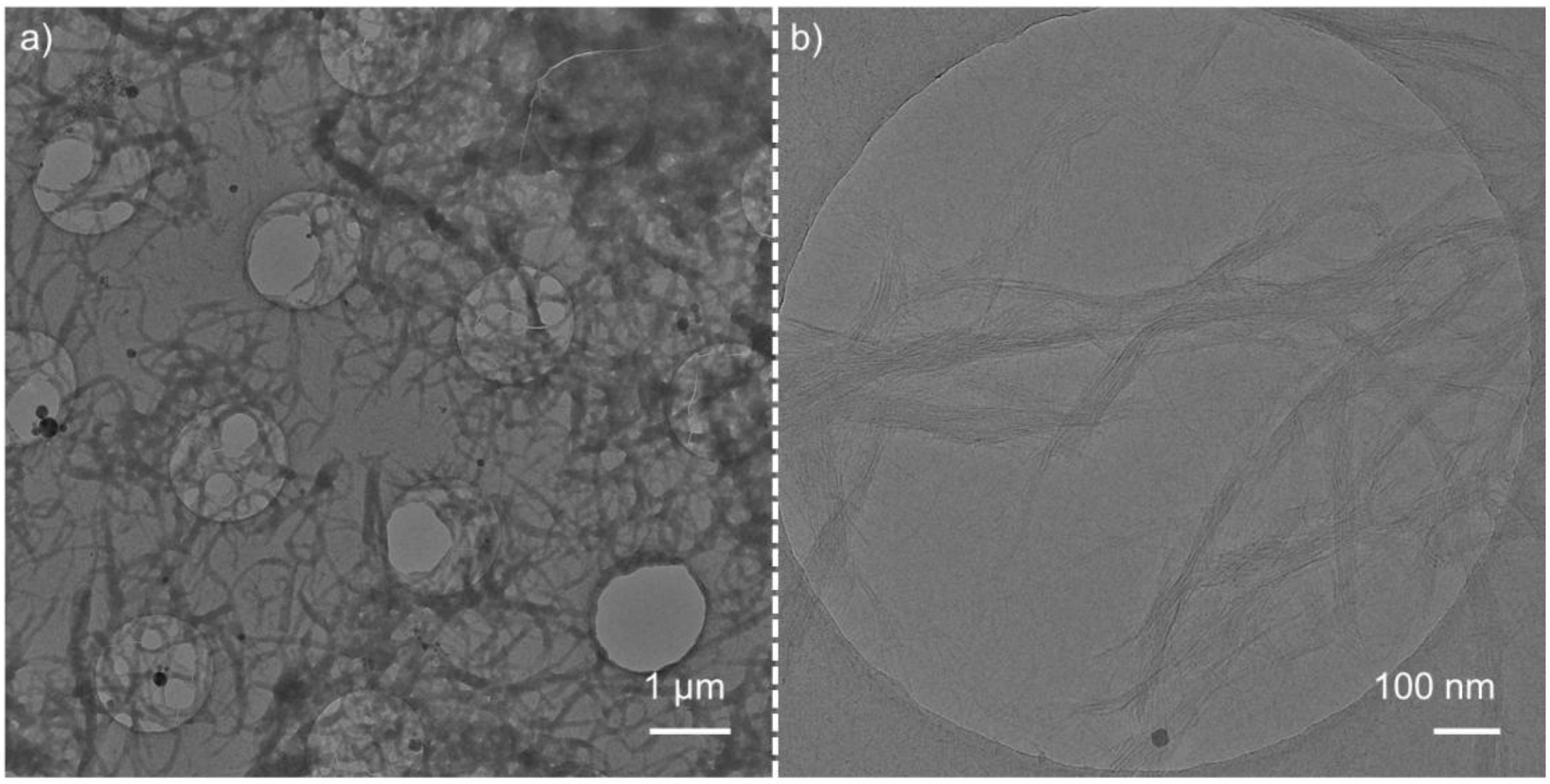

Figure S15: Cryo-TEM images at a) 3400x and b) 28000x magnification of $\mathbf{A^m}$ in water vitrified at 36 °C.

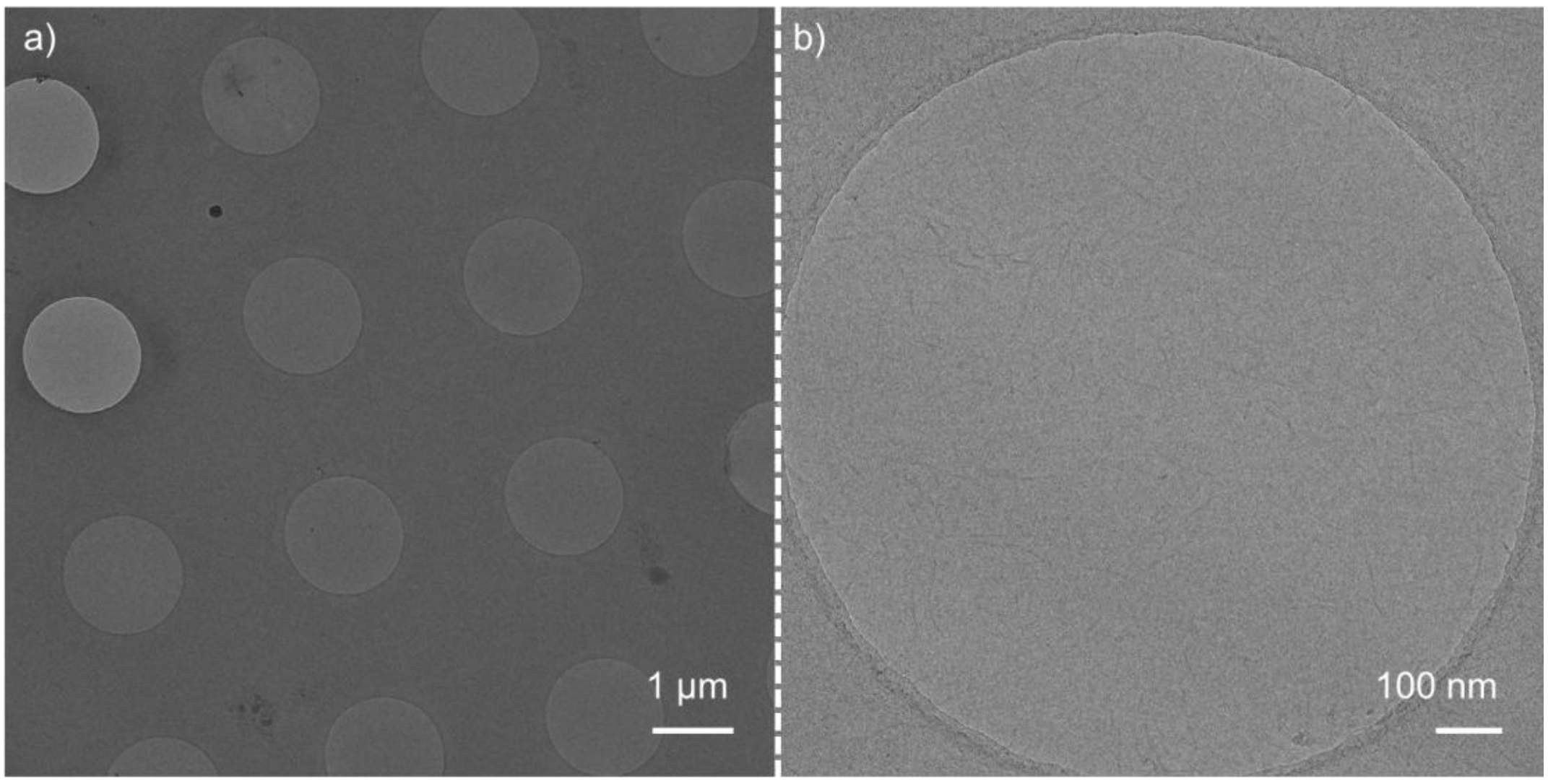

Figure S16: Cryo-TEM images at a) 3400x and b) 28000x magnification of **A**^{p} in water vitrified at 10 °C.

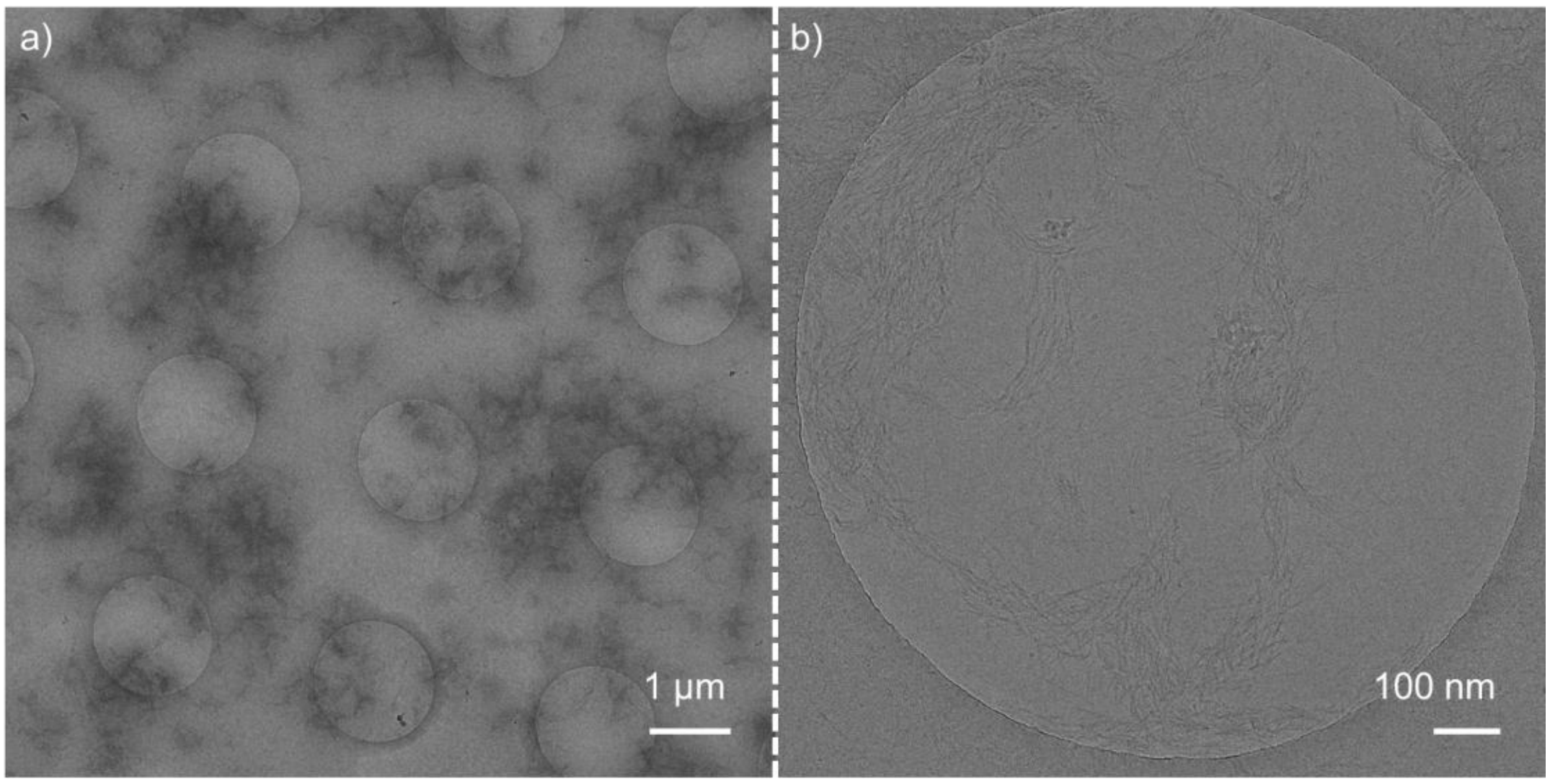

Figure S17: Cryo-TEM images at a) 3400x and b) 28000x magnification of **A**^{p} in water vitrified at 36 °C.

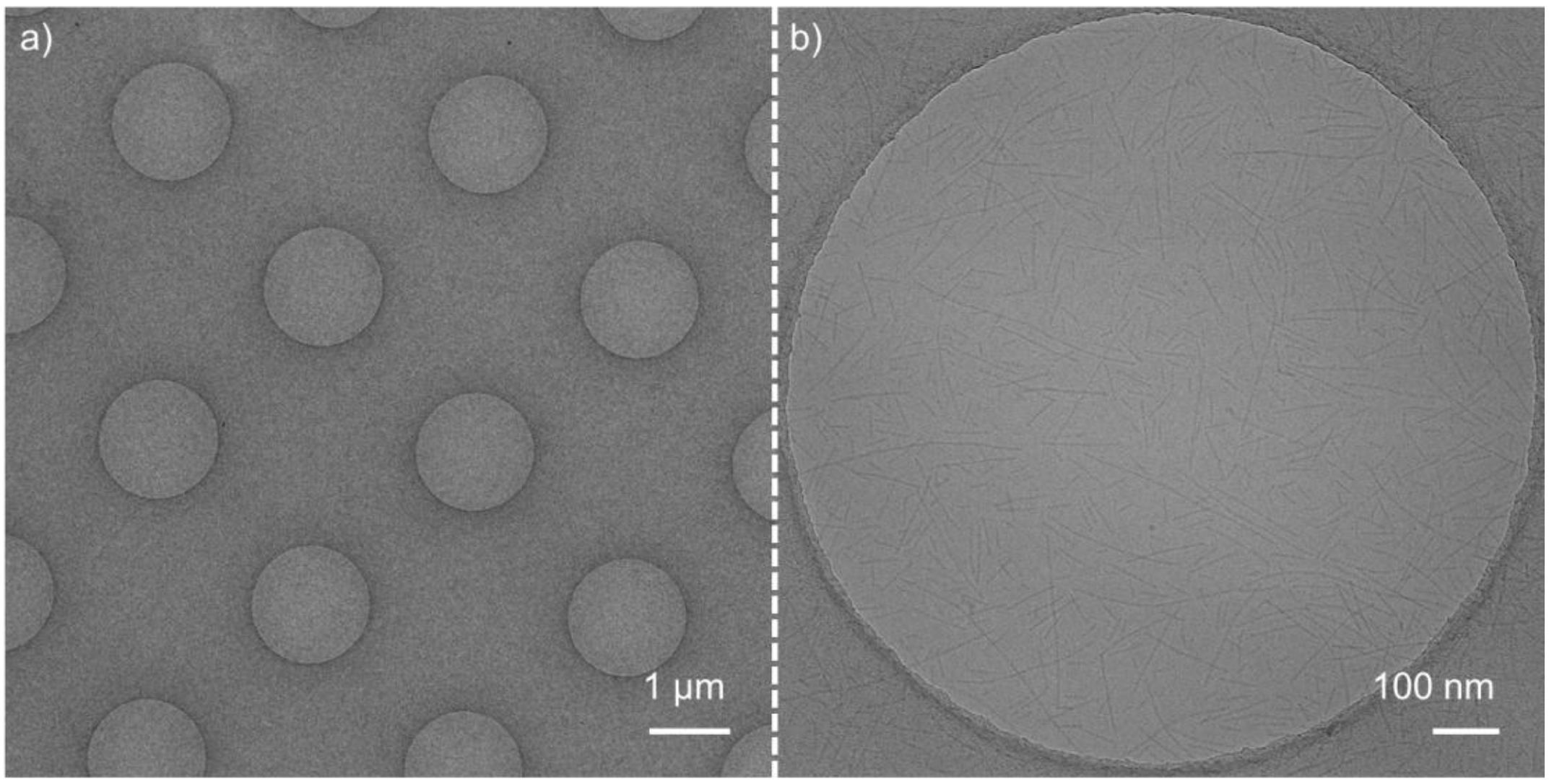

Figure S18: Cryo-TEM images at a) 3400x and b) 28000x magnification of **A°** in 0.5 M NaCl vitrified at 10 °C.

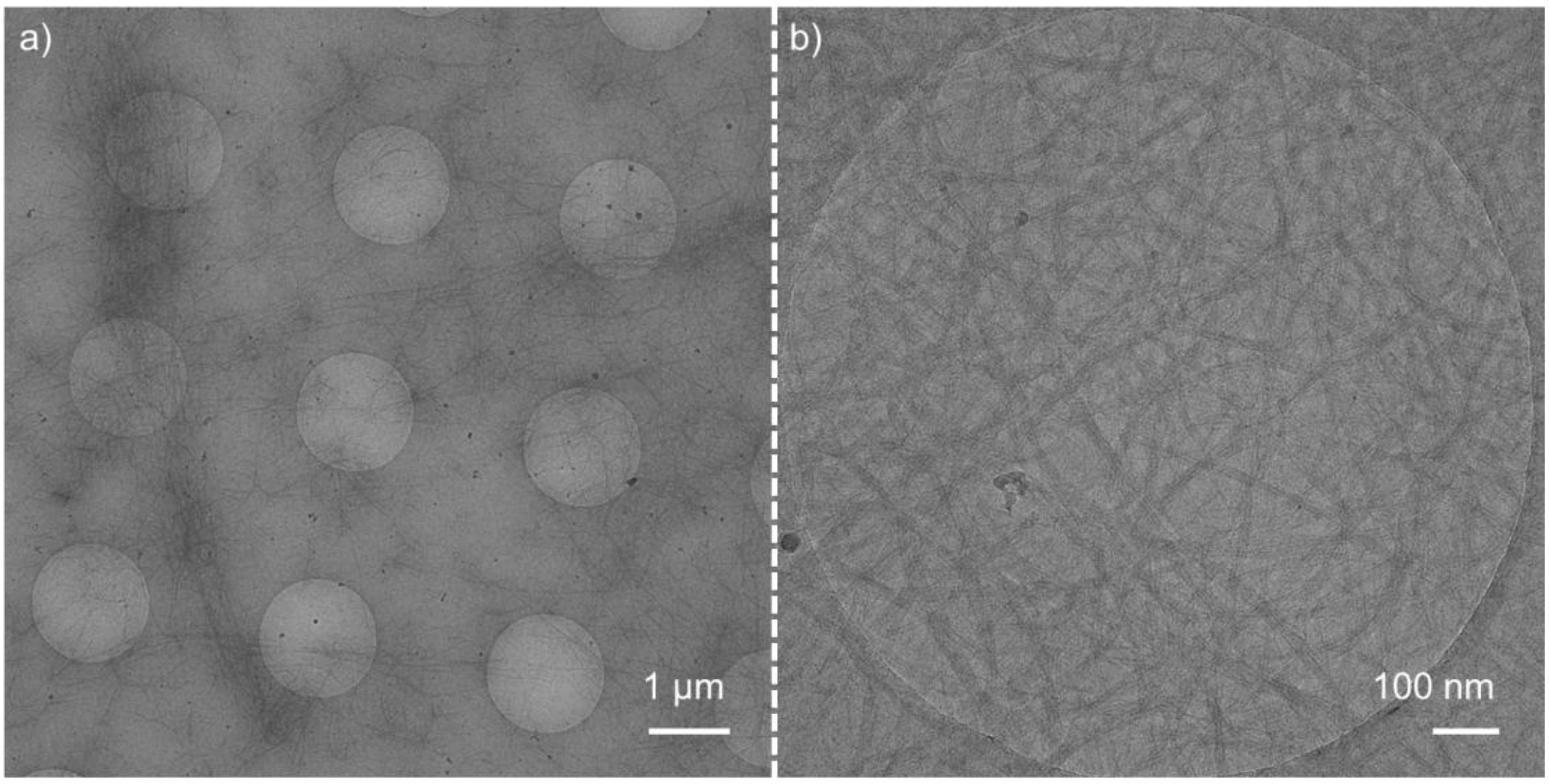

Figure S19: Cryo-TEM images at a) 3400x and b) 28000x magnification of **A°** in 0.5 M NaCl vitrified at 26 °C.

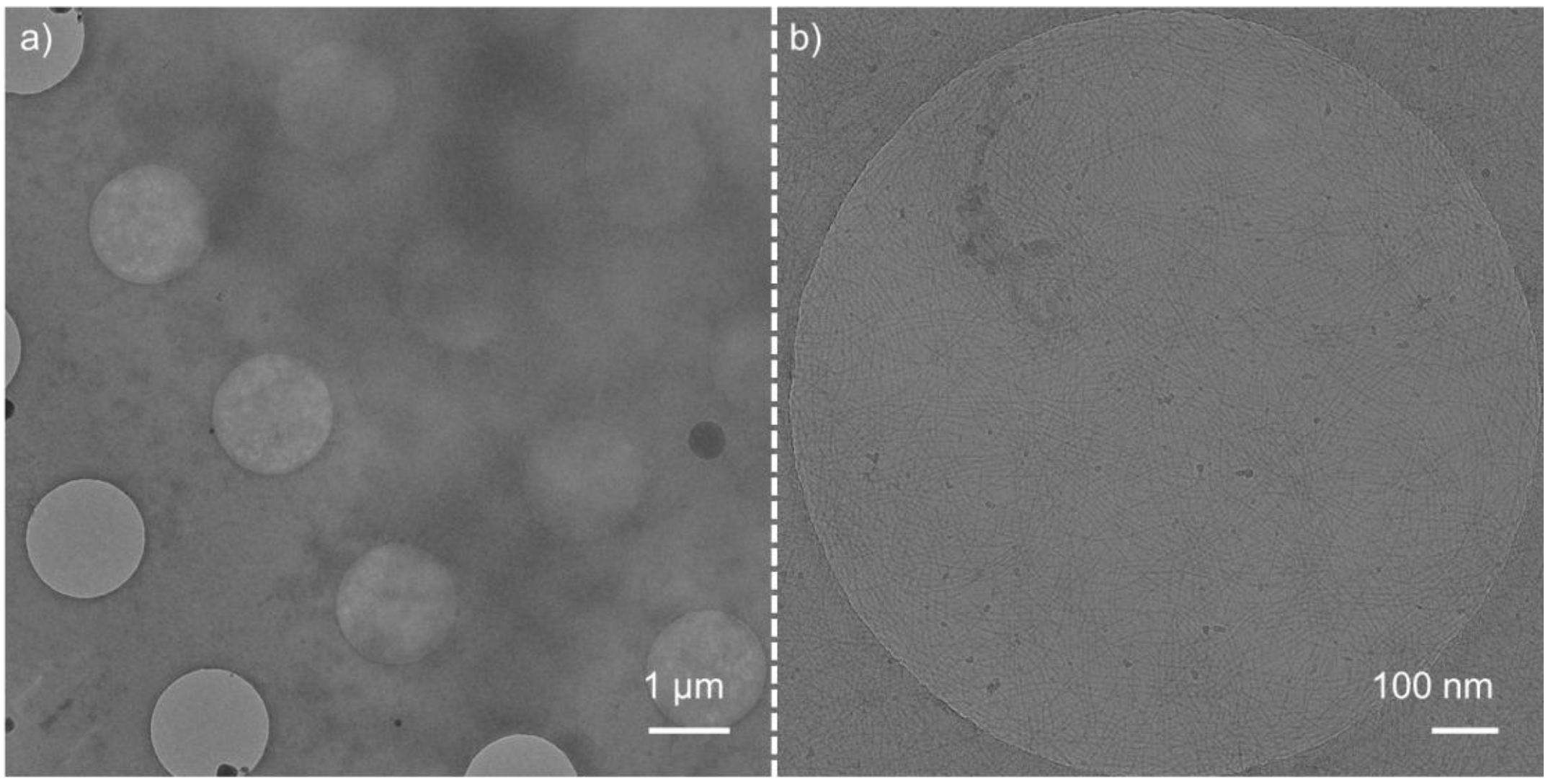

Figure S20: Cryo-TEM images at a) 3400x and b) 28000x magnification of **A°** in 15 % EtOH vitrified at 28 °C.

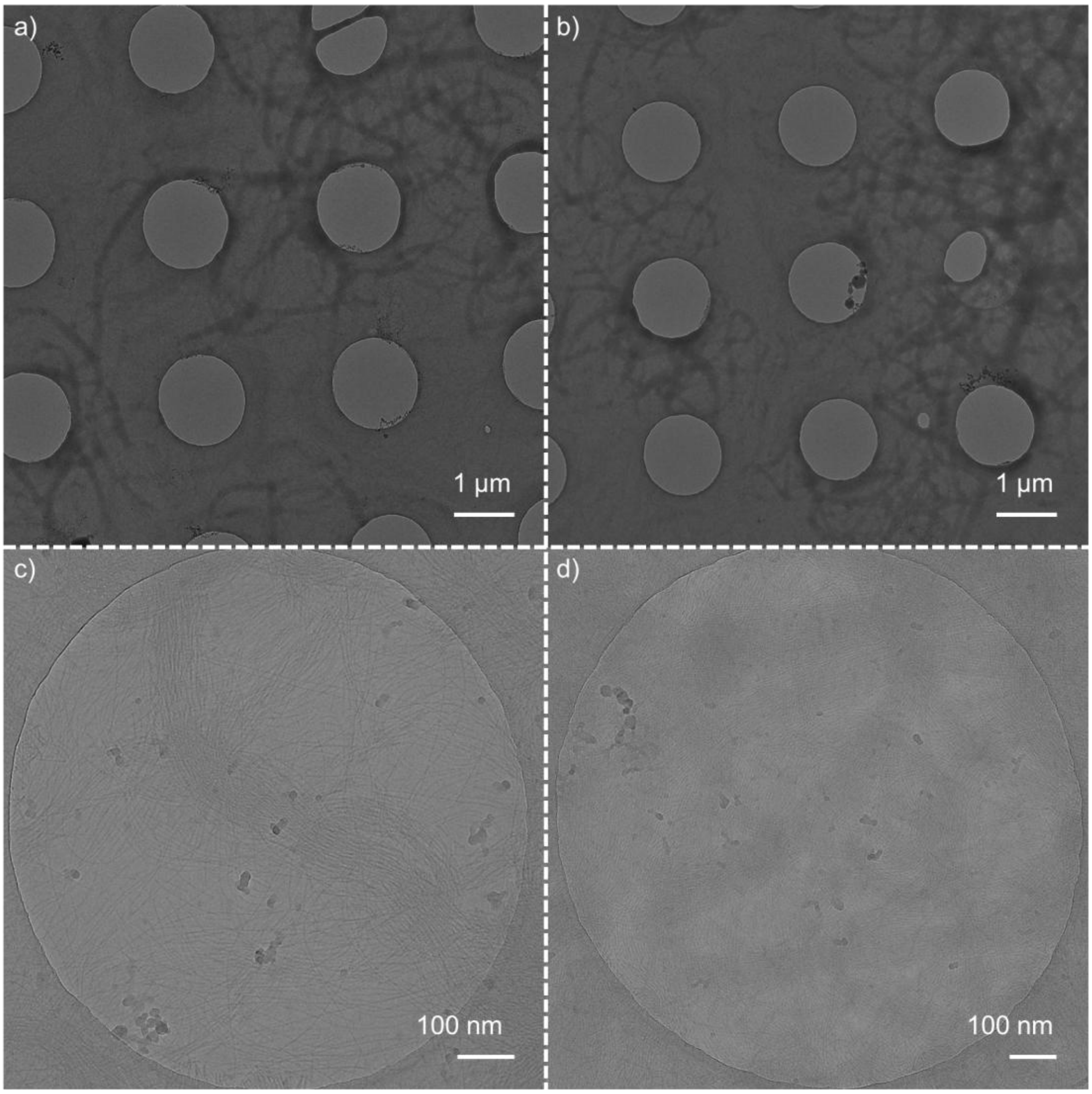

Figure S21: Cryo-TEM images at a-b) 3400x and c-d) 28000x magnification of a 1:1 mixture of **A°** and **B°** in water vitrified at 36 °C.

S04 Fluorescence Lifetime Imaging Microscopy

Sample preparation protocol: 1 mM stock solutions were prepared by dissolving the polymers in either MQ water or abs. EtOH using prolonged shaking or ultrasonication. The 0.1 mM solutions were obtained through 10-fold dilution. Before lifetime measurements, the two stock solutions with identical compounds and concentrations were mixed in the desired ratio in an Eppendorf tube (total volume of mixture was 200 µL). The mixtures were vigorously shaken for 1-2 minutes to redissolve the polymer that precipitated upon the liquid phases coming in contact. In all the cases of macroscopic precipitation under co-non-solvency conditions (~20-40 vol% EtOH content), the precipitate adhered to the walls of the Eppendorf tube, and the "clear" supernatant was transferred to the measurement cell.

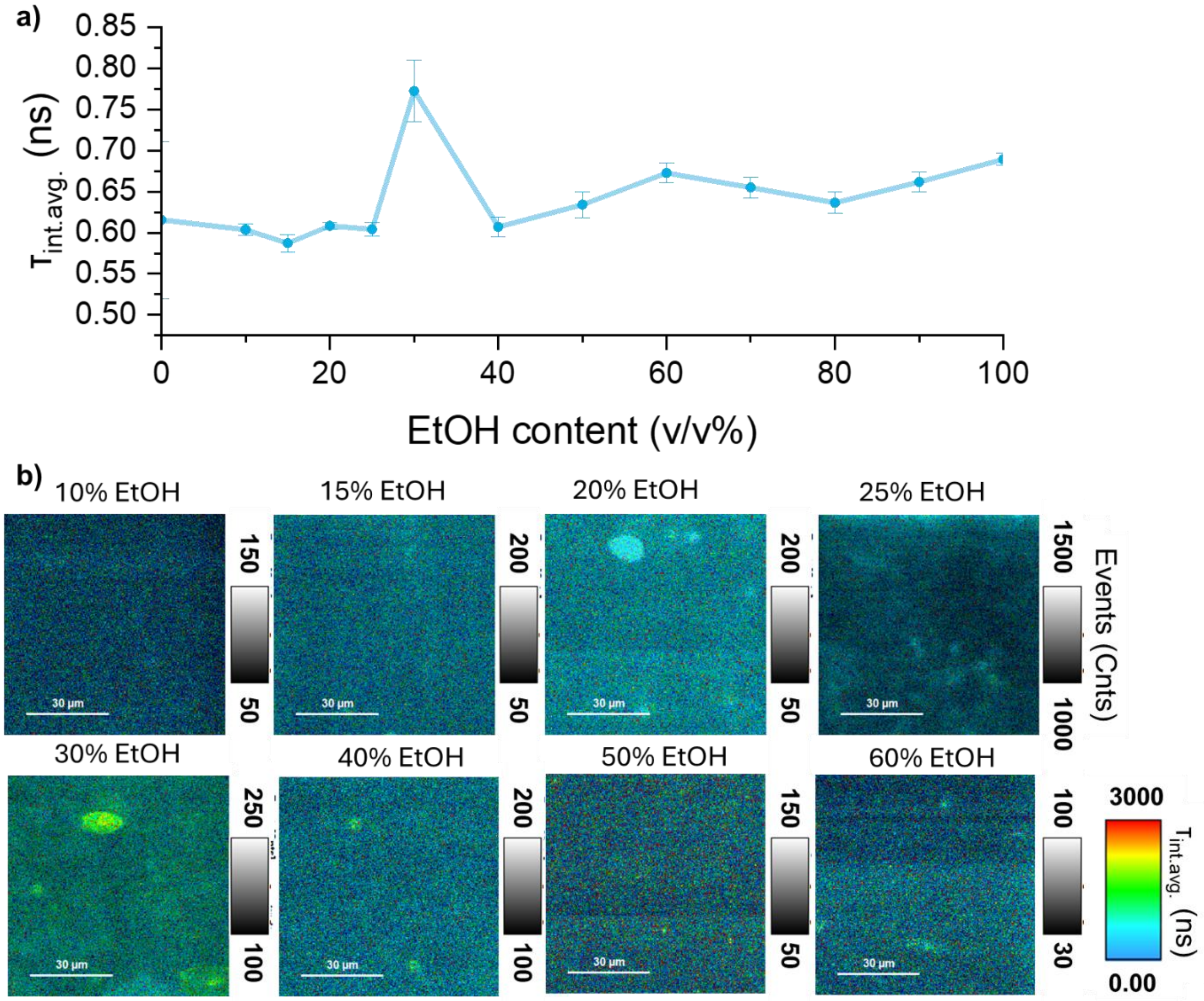

Figure S22: a) Fluorescence lifetime (3 parameter arbitrary fitting; $\lambda_{exc.}$=405, λ_{cutoff}<425 nm) and (b) FLIM lifetime (blue-to-red) and fluorescence intensity (grayscale) heatmaps of 0.1 mM **A°** in water/EtOH mixtures with ethanol contents ranging from 15 to 60 vol%.

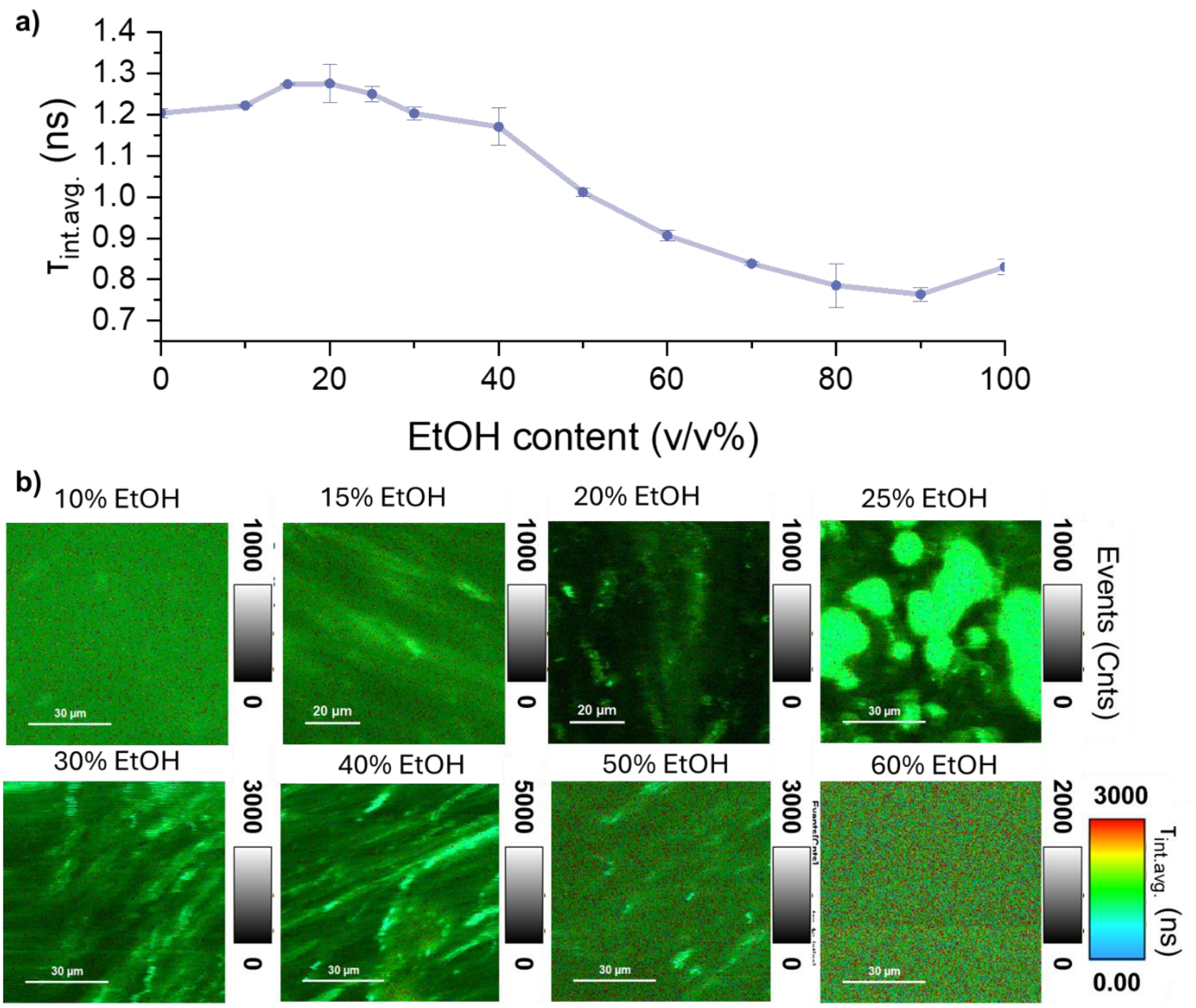

Figure S23: a) Fluorescence lifetime (3 parameter arbitrary fitting; $\lambda_{exc.}$=405, λ_{cutoff}<425 nm) and (b) FLIM lifetime (blue-to-red) and fluorescence intensity (grayscale) heatmaps of 0.1 mM **A**^{m} in water/EtOH mixtures with ethanol contents ranging from 15 to 60 vol%.

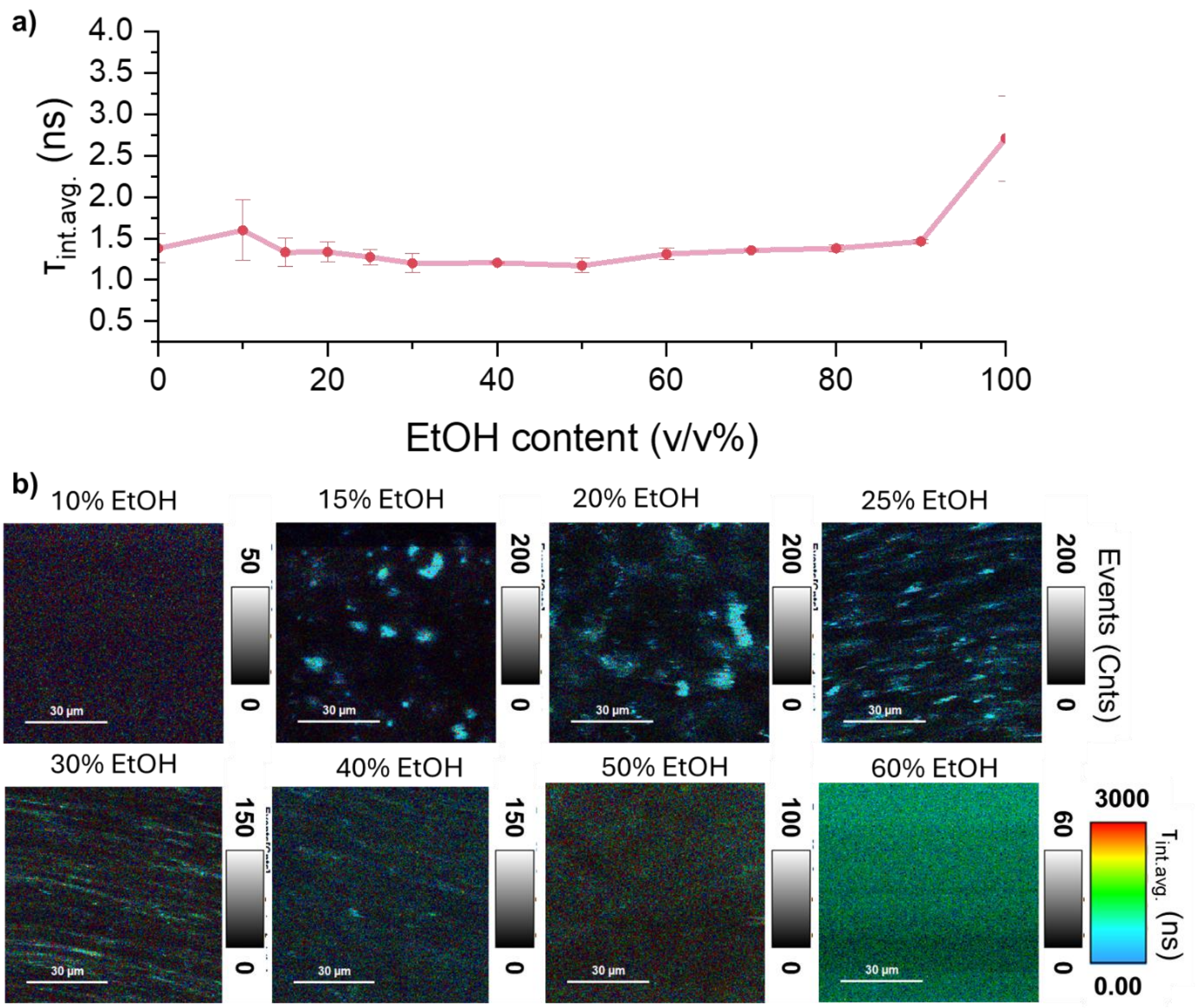

Figure S24: a) Fluorescence lifetime (3 parameter arbitrary fitting; $\lambda_{exc.}$=405, λ_{cutoff}<425 nm) and (b) FLIM lifetime (blue-to-red) and fluorescence intensity (grayscale) heatmaps of 0.1 mM **A^p^** in water/EtOH mixtures with ethanol contents ranging from 15 to 60 vol%.

S05 Rheology

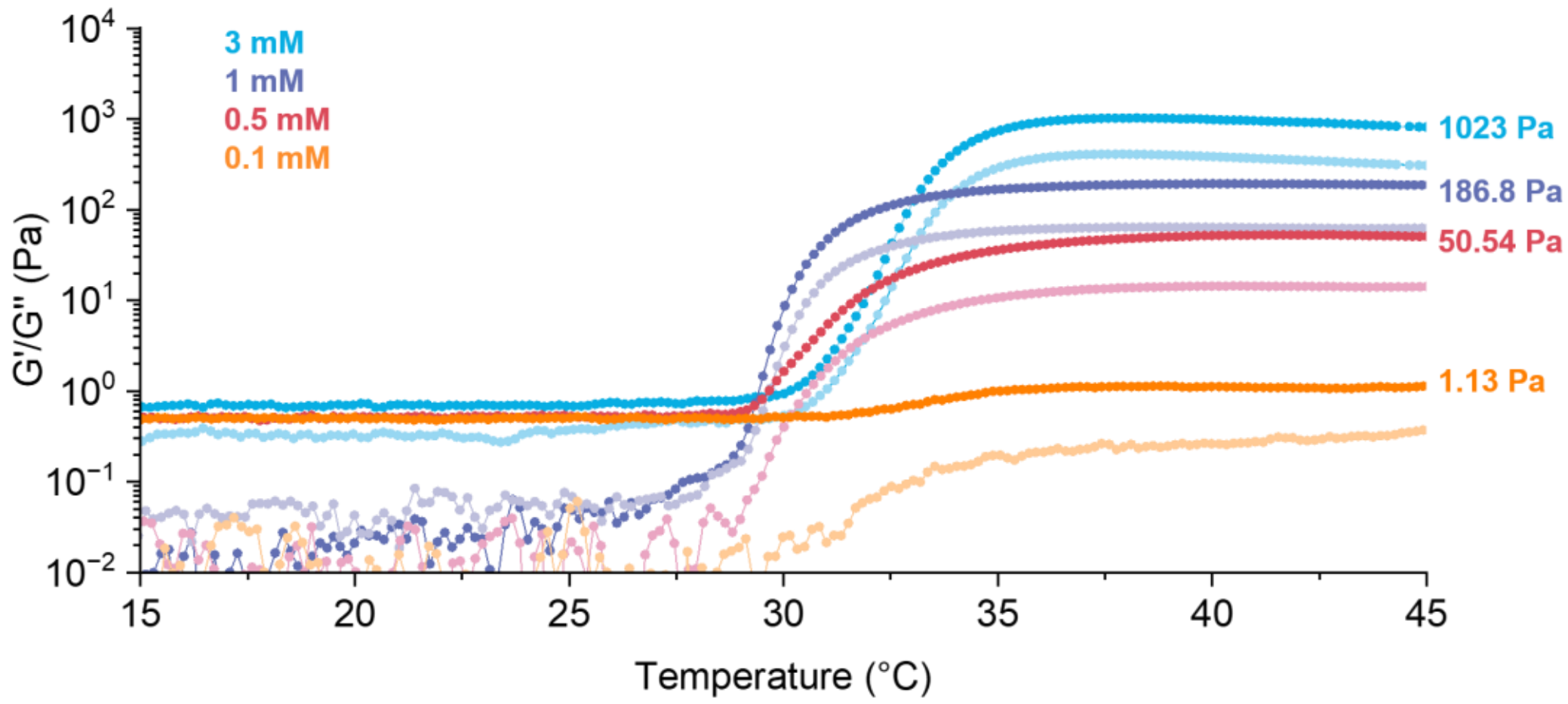

Figure S25: Rheology heating curves (*G'* strong, *G''* pale) of solutions of **A°** in water of varying concentrations.

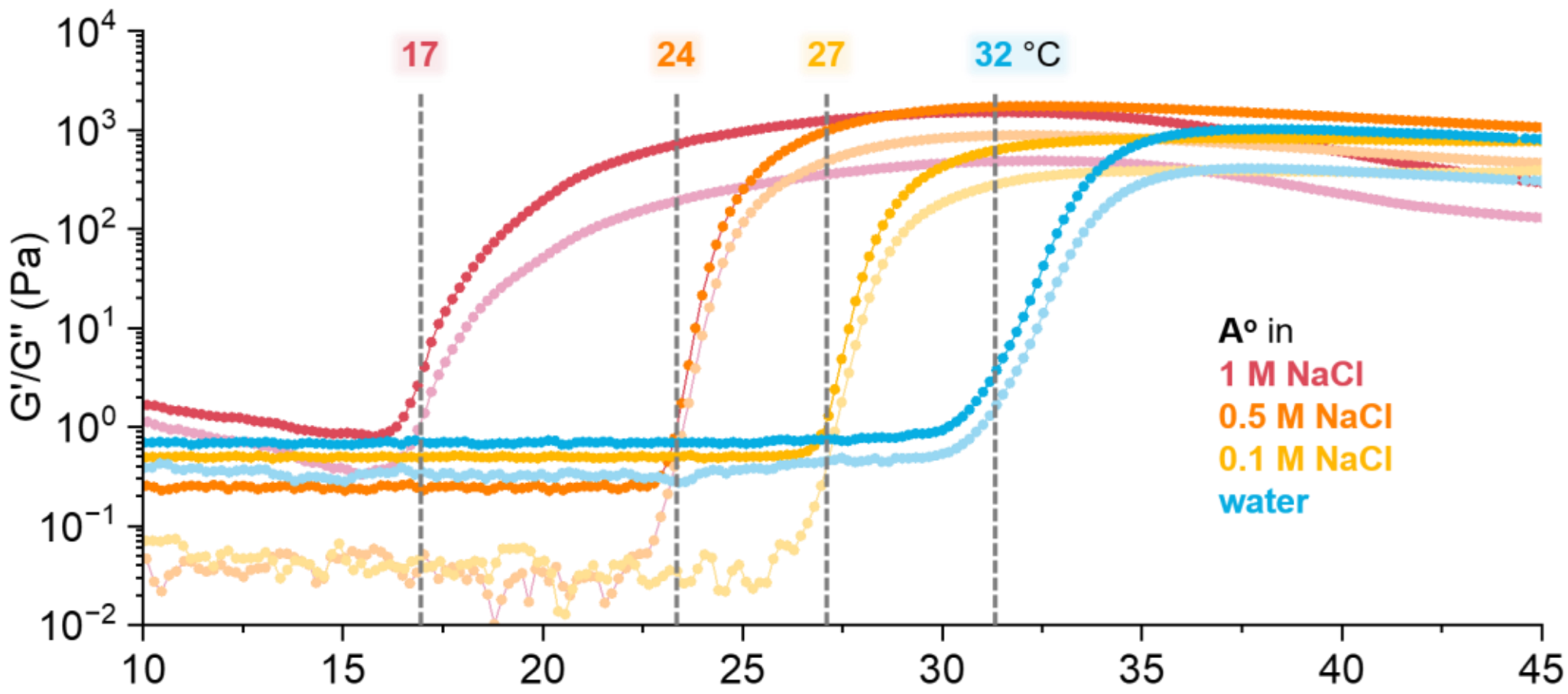

Figure S26: Rheology heating curves (*G'* strong, *G''* pale) of 3 mM **A°** in pure water and aqueous NaCl solutions of varying concentrations.

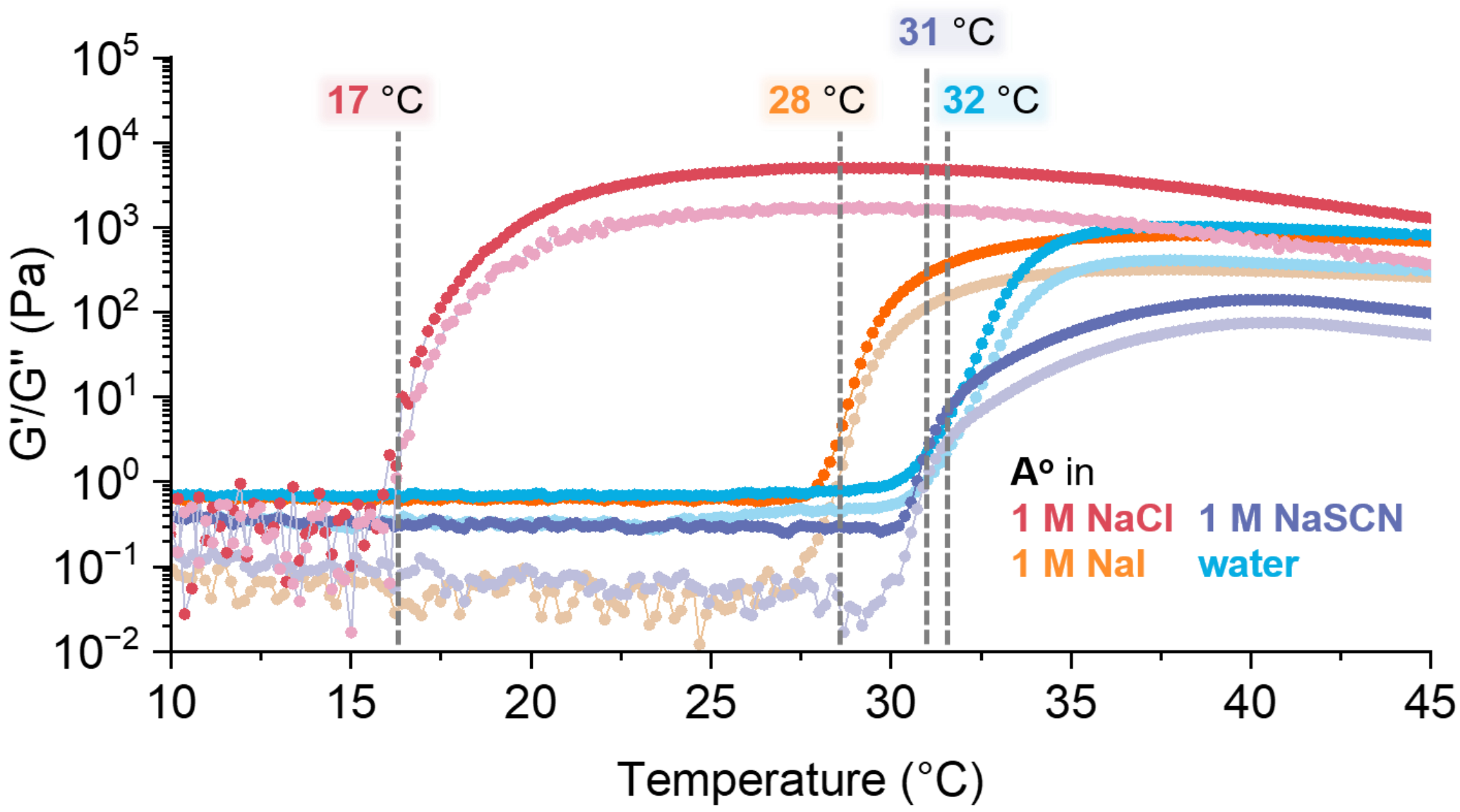

Figure S27: Rheology heating curves (*G'* strong, *G''* pale) of 3 mM **A°** in 1 M aqueous NaCl, NaI and NaSCN as well as pure water, respectively.

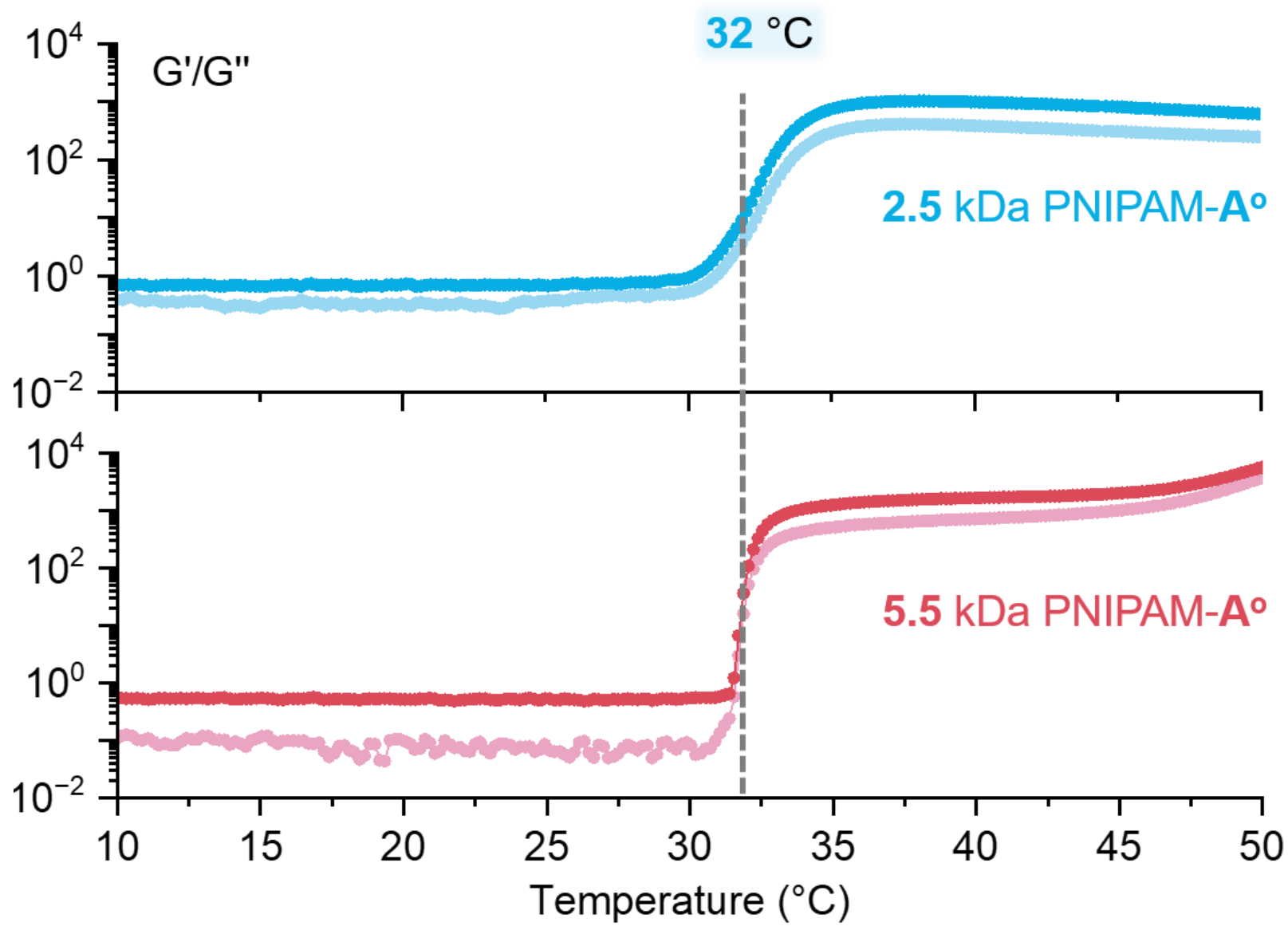

Figure S28: Rheology heating curves (*G'* strong, *G"* pale) of 3 mM 2.5 kDa PNIPAM-**A°** (top) and 5.5 kDa PNIPAM-**A°** (bottom) in pure water and aqueous NaCl solutions of varying concentrations.

S06 Transmetallation with Ni(II)

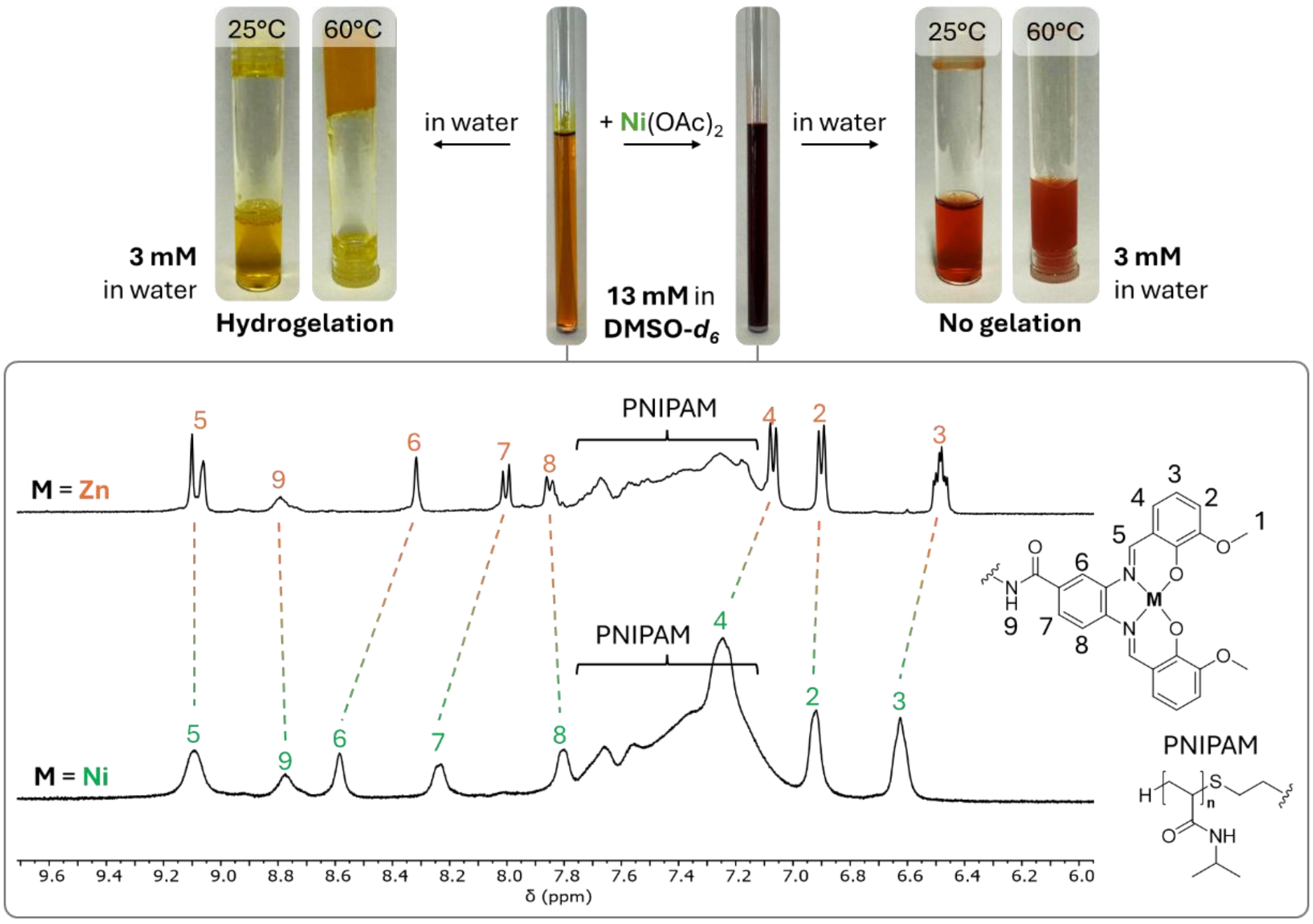

Figure S29: Photographs of solutions of the NMR sample of **A°** in DMSO-d_6 (13 mM) before and after the addition of $Ni(OAc)_2$ as well as the resulting 3 mM aqueous solutions after dilution of 230 µL of NMR sample into 1 mL water showing hydrogelation in the absence of $Ni(OAc)_2$ and no gelation after Zn-Ni exchange. Corresponding ^{1}H NMR (400 MHz, DMSO-d_6) spectra show significant shifts of the signals in the aromatic region upon transmetallation.

S07 Photothermally Induced Hydrogelation Experiments

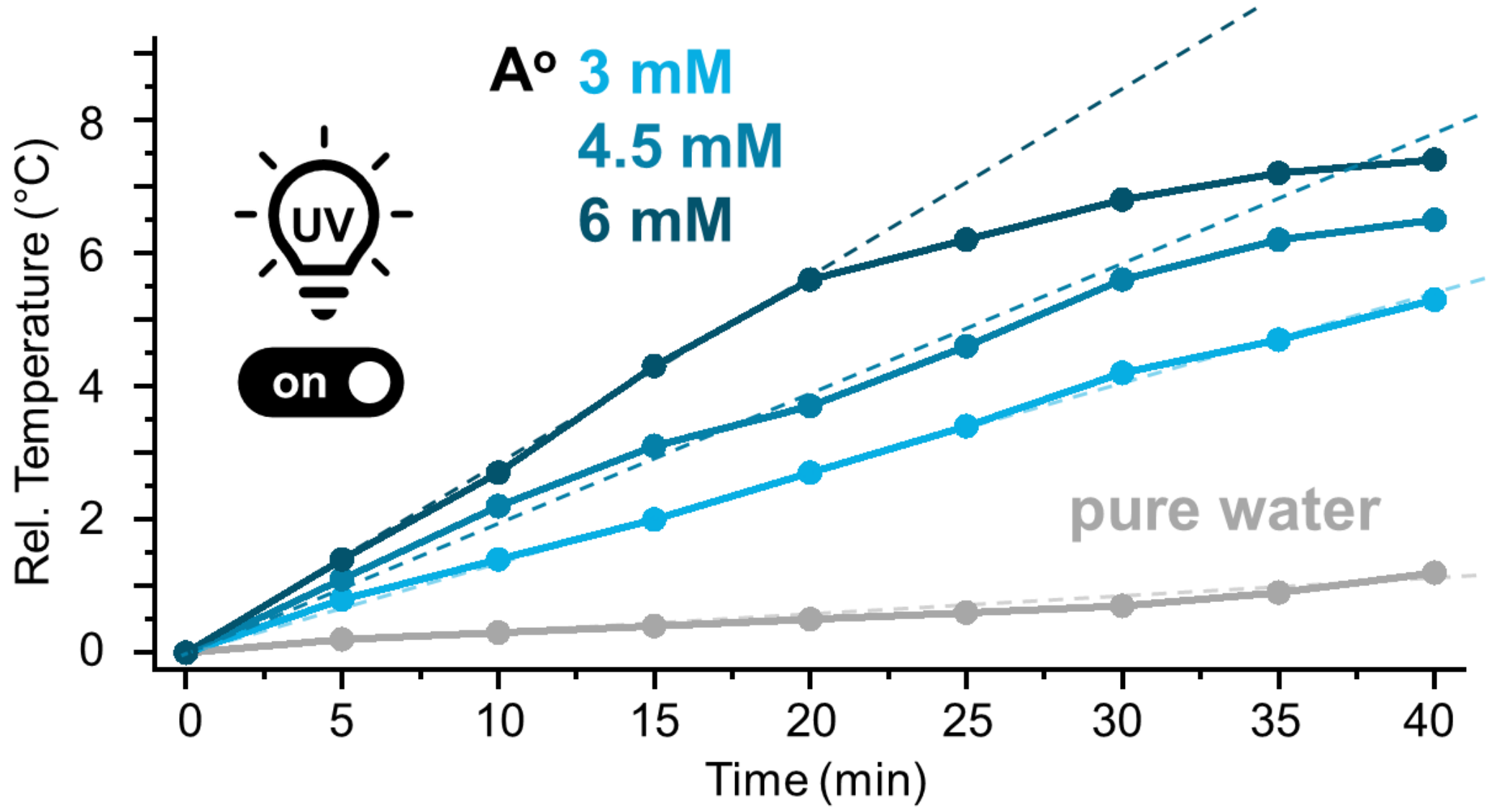

Figure S30: Photothermally induced relative temperature change by UV irradiation of aqueous solutions of **Aº** of varying concentration and pure water for reference (1.5 mL in 10 mm x 10 mm Quartz cuvette). The direct comparison highlights the neglectable temperature change of pure water without the presence of chromophores.

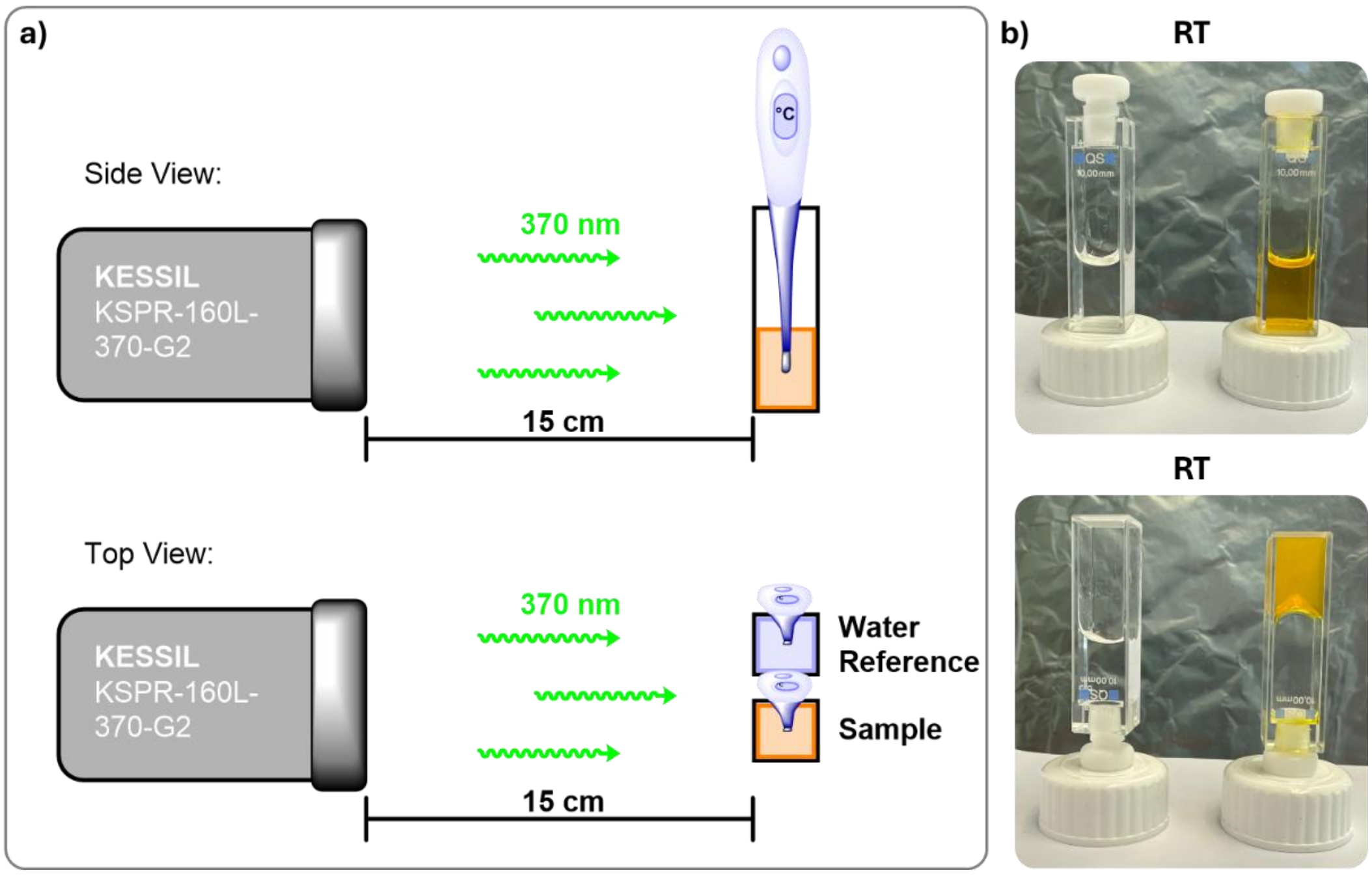

Figure S31: a) Schematic representation of photothermally induced hydrogelation experiments. b) Digital photographs of quartz cuvettes containing a 3 mM aqueous solution of **Aº** and pure water for reference before (top) and after (bottom) irradiation.

S08 References

[1] M. R. Stühler, H. Makki, T. Hilal, D. Chakraboty, M. Dimde, K. Ludwig, R. Haag, S. Rosenfeldt, D. Silbernagl and A. Schäfer, *Adv. Mater.*, 2026, e19933.

[2] H. Lai, T. Zhao, Y. Deng, C. Fan, W. Wu and C. Yang, *Chin. Chem. Lett.*, 2019, **30**, 1979-1983.
